# Dendritic targeting of short and long 3′ UTR BDNF mRNA is regulated by BDNF or NT-3 and distinct sets of RNA-binding proteins

**DOI:** 10.3389/fnmol.2015.00062

**Published:** 2015-10-29

**Authors:** Annalisa Vicario, Andrea Colliva, Antonia Ratti, Laetitia Davidovic, Gabriele Baj, Łukasz Gricman, Claudia Colombrita, Alberto Pallavicini, Kevin R. Jones, Barbara Bardoni, Enrico Tongiorgi

**Affiliations:** ^1^Department of Life Sciences, University of TriesteTrieste, Italy; ^2^Laboratory of Neuroscience – IRCCS Istituto Auxologico ItalianoMilano, Italy; ^3^Department of Pathophysiology and Transplantation, “Dino Ferrari Center", Università degli Studi di MilanoMilano, Italy; ^4^INSERM, IPMC – CNRS, UMR 7275Valbonne, France; ^5^Department of Molecular Cellular and Developmental Biology, University of Colorado, BoulderCO, USA

**Keywords:** neurotrophins, brain-derived neurotrophic factor, dendrites, hippocampus, RNA transport, RNA binding proteins, Fragile-X

## Abstract

Sorting of mRNAs in neuronal dendrites relies upon inducible transport mechanisms whose molecular bases are poorly understood. We investigated here the mechanism of inducible dendritic targeting of rat brain-derived neurotrophic factor (BDNF) mRNAs as a paradigmatic example. BDNF encodes multiple mRNAs with either short or long 3′ UTR, both hypothesized to harbor inducible dendritic targeting signals. However, the mechanisms of sorting of the two 3′ UTR isoforms are controversial. We found that dendritic localization of BDNF mRNAs with short 3′ UTR was induced by depolarization and NT3 *in vitro* or by seizures *in vivo* and required CPEB-1, -2 and ELAV-2, -4. Dendritic targeting of long 3′ UTR was induced by activity or BDNF and required CPEB-1 and the relief of soma-retention signals mediated by ELAV-1, -3, -4, and FXR proteins. Thus, long and short 3′ UTRs, by using different sets of RNA-binding proteins provide a mechanism of selective targeting in response to different stimuli which may underlay distinct roles of BDNF variants in neuronal development and plasticity.

## Introduction

**FIGURE 1:**
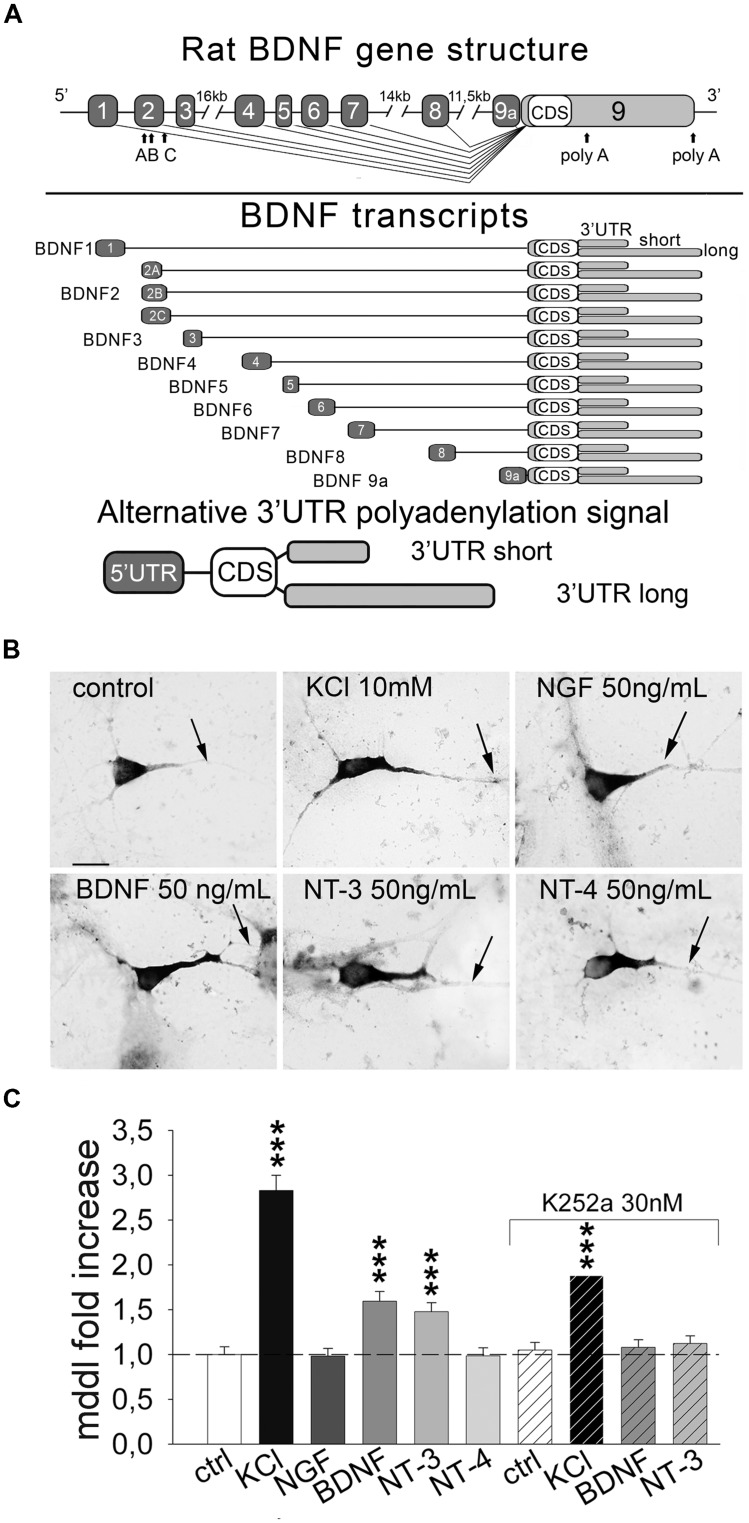
**Inducible targeting of endogenous *BDNF* mRNA in primary hippocampal neurons.** **(A)** Upper panel, illustration of the rat BDNF gene structure with the 5′ UTR exons (light gray) and the coding sequence/3′ UTRs exon (black); bottom panel, a schematic representation of the rat splice variants according to the nomenclature proposed by Aid et al. (2007). **(B)** Representative *in situ* hybridization (ISH) on control cultures and after 3 h of 10 mM KCl, 50 ng/mL of NGF, BDNF, NT-3 and NT-4 stimulation; scale bar 10 μm. **(C)** Quantification of *BDNF* mRNA maximum dendritic distance labeling (MDDL) in dendrites of treated neurons normalized to control neurons upon different treatments with or without Trk inhibitor K252a (^∗∗∗^*P* < 0.001 vs. control, Kruskal–Wallis one way ANOVA on Ranks followed by Dunn’s multiple comparison test).

Subcellular compartmentalization of mRNAs is a mechanism enabling local synthesis of proteins required for neuronal development and plasticity such as brain-derived neurotrophic factor (BDNF; Tongiorgi et al., 1997; Steward and Schuman, 2001; Soule et al., 2006; Rodriguez et al., 2008; Tongiorgi, 2008; Martin and Ephrussi, 2009). Although *BDNF* mRNA abundance in dendrites is generally low, dendritic localization is enhanced in response to membrane depolarization (Tongiorgi et al., 1997) or to BDNF itself (Righi et al., 2000). Evidence that *BDNF* mRNA translation can occur in dendrites was provided by studies *ex vivo* using neurosynaptosomes (Raju et al., 2011; Gharami and Das, 2014) and *in vitro* in hippocampal neurons (An et al., 2008; Verpelli et al., 2010; Baj et al., 2011). The mechanism underlying *BDNF* mRNA targeting to dendrites is poorly understood, partly because of the complexity of *BDNF* gene regulation (**Figure 1A**, upper panel). Transcription of rodent *BDNF* gene produces 11 transcripts, each with a different alternatively spliced 5′ untranslated region (5′ UTR) and the same coding region (CDS; **Figure 1A**; Aid et al., 2007; Pruunsild et al., 2007). These transcripts can exist in two isoforms, either with long (3.2 Kb) or short (0.3 Kb) 3′ UTR (**Figure 1A**, bottom panel; Aid et al., 2007; Pruunsild et al., 2007). There is general consensus that most *BDNF* transcripts are restricted to the soma and proximal dendrites (e.g., exons 1, 4) but selected variants are transported distally (e.g., exons 2, 6) in an activity-dependent manner (An et al., 2008; Chiaruttini et al., 2008, 2009; Aliaga et al., 2009; Baj et al., 2013; Will et al., 2013).

To explain the mechanism of BDNF transcripts’ sorting, two alternative models were proposed. The first is a dualistic mechanism by which somatic *BDNF* transcripts express the short 3′ UTR while dendritic transcripts have the long 3′ UTR (An et al., 2008). However, in rodent brain models, transcripts containing exons 1, 2, 4, and 6 (formerly I, II, III, IV) are expressed with long or short 3′ UTR in a 1:1 ratio and, after kainic acid-induced seizures, transcripts having mainly the short 3′ UTR are detected (Timmusk et al., 1993). Remarkably, following seizures, exons 2 and 6 were detected in distal dendrites (Chiaruttini et al., 2008; Baj et al., 2013). Our alternative model is based on a tripartite combinatorial mechanism in which “selector” signals are located in the 5′ UTR (for dendrite or soma destination), a constitutively active dendritic targeting element (DTE) mediated by Translin in the CDS, and activity-dependent DTEs harbored in both long and short 3′ UTRs (Pattabiraman et al., 2005; Chiaruttini et al., 2008, 2009; Aliaga et al., 2009; Baj et al., 2011, 2013). In support of this model, *BDNF* mRNAs with either 3′ UTRs were shown to be transported in dendrites in response to neural activity (Oe and Yoneda, 2010; Baj et al., 2011). Activity-dependent dendritic targeting of the 3′ UTR short requires the RNA-binding protein (RBP) CPEB-1 (Oe and Yoneda, 2010) while the targeting mechanism of the 3′ UTR long was not investigated. Here, we investigated the dendritic targeting mechanism of *BDNF* transcripts with long or short 3′ UTR, demonstrating selective induction by BDNF or NT-3, respectively thanks to interactions with distinct sets of RBPs.

## Materials and Methods

### Chimaeric GFP Constructs

Total RNA was extracted from whole rat brain using TriZol^®^ Reagent (Invitrogen). 1 μg of total RNA was reverse-transcribed into cDNA and amplified with specific primer pairs. For details on BDNF CDS-GFP chimera cloning (please see, Chiaruttini et al., 2009). PCRs were performed with Phusion Hi-Fidelity DNA polymerase (New England BioLabs) using specific primer pairs and PCR conditions (reported in **Supplementary Table S1**). A direct amplification and cloning in pEGFP-N1 vector (Clontech) was performed for BDNF 3′ UTRshort, 3′ UTRshort-mutELAV-up, 3′ UTRlong, 3′ UTRmid and 3′ UTRend. For 3′ UTRshort-mutCPE and 3′ UTRshort-mutELAV-dw cloning, an assembly strategy was adopted. Briefly a first round of PCR was performed using the primer pairs for CPE and ELAV-dw mutations (**Supplementary Table S1**). The second round of PCR was performed using CPE and ELAV-dw mutant fragments obtained in the first round as forward “megaprimers” with 3′ UTRsh reverse primer to obtain mutated full length 3′ UTR short sequences. PCR fragments were then purified (PCR Cleanup kit from Sigma-Aldrich) and digested with XhoI/SacII for BDNF coding sequence or NotI/HpaI for 3′ UTRs mutants. All restriction enzymes were purchased from New England BioLabs. Digested fragments were gel purified (PCR Gene Elute kit from Sigma-Aldrich) and ligated (T5 DNA ligase from New England BioLabs) over night at 16° with properly digested pEGFP-N1 vector.

### Protocol for CLIP

The CLIP (cross-linking and immunoprecipitation) assay for ELAV was performed as previously described by Ratti et al. (2006) and for FMRP was carried out using brains lysates from wild type (WT) and *Fmr1*-KO mice as described by Davidovic et al. (2011). Radiolabeled riboprobes for BDNF 3′ UTR sequences were obtained by transcribing pBluescript KS containing BDNF 3′ UTR short, 3′ UTR mid, and 3′ UTR end sequences. Oligos and conditions used for BDNF coding sequence Reverse transcriptase-PCR on CLIP are reported in **Supplementary Table S1**. PCR oligos and conditions used for Pgk1 and Map1b were previously described by Edbauer et al. (2010).

### Bioinformatic Analysis

Analysis of evolutionary conserved regions in BDNF CDS and 3′ UTRs was performed using phastCons alignment software (Siepel et al., 2005). The research of putative *cis*-elements for RBPs on rat BDNF 3′ UTR sequence was performed using Bioedit software (http://www.mbio.ncsu.edu/BioEdit/bioedit.html). The RNA motifs reported in literature for the cited RBPs were pairwise aligned on the target sequence and at least 80% homology was set as threshold to define a putative *cis*-element.

### RNAi Interference

RNAi interference (RNAi) “cocktails” against the different RBPs were generated by RNAseIII (Ambion) cleavage of relatively long (∼300 nt) double-stranded RNA. Target regions of mRNA encoding for RBPs were identified by consulting the NCBI data base. To amplify the target regions, oligonucleotidic primers containing the T7 promoter were used to amplify the target regions and produce siRNA following manufacturer instructions. Sequences and protocol are reported in **Supplementary Table S2**. In order to evaluate RNAi “cocktails” efficacy and specificity, reverse transcriptase PCR was performed on RNA extracted from primary hippocampal cultures of rat neurons. To amplify the targeted mRNAs we used oligonucleotidic primers containing the same sequence designed for siRNA pools generation. Sequences and protocol are reported in **Supplementary Table S2**. For quantification, PCR bands were scanned and quantified by the Quantity One^®^ 4.6.6. software (Bio-Rad, Hercules, CA, USA).

### Cell Cultures

Primary rat and mouse hippocampal neurons were prepared from P0 pups as previously described (Tongiorgi et al., 1997) with slight modifications. Cells were plated on 2% Matrigel (BD Biosciences) coated coverslips in 24-well plates at a density of 2 × 10^5^ cells per well and cultured for 6 days in neurobasal supplemented with B27 (Invitrogen) and pen/strep in a 5% CO_2_-humidified incubator. The medium was changed every 2 days from the second day in culture onward. PC12 rat pheochromocytoma cells, used to asses RBP siRNAs efficiency and specificity, were cultured in RPMI medium supplemented with 10% FBS, 5% Donor Horse Serum, 2 mM L-glutamine, 20 mM Hepes and antibiotics (Euroclone) and differentiated in DMEM containing 1% horse serum, 1% Pen-Strep, and 50 ng/ml NGF (Sigma). Cells were transfected with Lipofectamine2000^TM^ (Invitrogen) with 1 μg of plasmid DNA or 19 nM of siRNA mixture (at 6 DIV for neurons or 75% confluency for PC12 cells). Neurons were depolarized as previously described (Tongiorgi et al., 1997), or treated with different neurotrophins (BDNF, NT-3, NT-4; SIGMA) at a 50 ng/mL concentration in culture medium. After 3 h of depolarization or neurotrophin administration, cells were fixed with PFA 4% for 15’ at RT.

### Animal Treatment

Animals were treated according to the institutional guidelines in compliance with the European Council Directive 86/609 and NIH Guide for the Care and Use of Laboratory Animals. Animal experiments on WT and BDNF^lox/lox^ mice were authorized by the Italian Ministero della Salute n. 185/2010-B d.d. 21/10/2010. Two month old male C57/BL6 or BDNF^lox/lox^ mice (Gorski et al., 2003) were anesthetized with urethane (1 g/kg, i.p.) 30 min prior to i.p. injection of 300 mg/kg pilocarpine or saline. Animals were housed in groups under standard animal room conditions (12:12 h light/dark cycle, ambient temperature 23°C, ad libitum access to food and water).

### *In situ* Hybridization and Fluorescent *In situ* Hybridization

*In situ* hybridization (ISH) on brain sections or cultures was performed as previously described (Tongiorgi et al., 1997, 1998). Cells were fixed for 15 min at RT in 4% PFA in PBS, washed in PBST, and permeabilized in absolute ethanol for 15 min at -20°C. After rehydration, cells were hybridized with antisense or sense probes for GFP mRNA or *BDNF* CDS. The open reading frame of GFP or BDNF CDS were subcloned into pBluescript or pGEM vectors respectively and DIG- labeled probes were synthesized with the DIG-RNA labeling kit (Roche Diagnostics) using linearized plasmids as templates, according to the manufacturer’s instructions. Hybridization was followed by high stringency washes with 0.01x Sodium Saline Citrate buffer containing 0.1% Tween20 (SSCT) at 60°C, then coverslips were incubated with anti-digoxigenin alkaline phosphatase coupled antibody and developed with NBT and BCIP for 40 min at RT. For fluorescent *in situ* hybridization (FISH) after PFA fixation cells were incubated with H_2_O_2_/PBS 0.3% for endogenous peroxydase quenching and then permeabilized with EtOH. After probe hybridization and stringency washes, coversplis were incubated with anti-digoxigenin horse-radish peroxidise conjugated (Roche) diluted 1:300 in blocking solution at RT for 1 h. HRP detection was performed using Tyramide System Amplification kit – Cyanin 3 coupled (Perkin Elmer) following manufacturer instructions. After three washes in TNT buffer, cells were incubated with the primary antibodies against different RBPs diluted in PBST/FBS 5% (**Supplementary Table S3**). Nuclei were stained with Hoechst 33342 and the coverslips were mounted with Mowiol (Sigma). ISH on free-floating, 40 μm coronal sections cut at the level of dorsal hippocampus was performed as previously described (Tongiorgi et al., 1997, 1998). After permeabilization, sections were pre-hybridized at 55°C for 1 h followed by an overnight incubation at 55°C with 100 ng/mL BDNF CDS antisense probe. ISH was stained with the fluorescent anti-DIG enhancer set (Roche diagnostics), following manufacturer’s instructions. Sections were mounted in anti-fade mounting solution (Invitrogen) and stored at 4°C in dark.

Confocal images of FISH on brain slices were acquired by a Nikon Eclipse C1si confocal system mounted on a Nikon TE-2000U inverted microscope. We used a Plan-Apochromat 20x/0.75(NA) and 60x/1.4(NA) oil-immersion objectives. The resolution used was of 0.2 μm × 0.2 μm × 0.5 μm (XxYxZ) for 20X acquisitions and 0.08 μm × 0.08 μm × 0.25 μm (XxYxZ) for 60X acquisitions. To obtain comparable signals between control and treated conditions, confocal images were acquired keeping the same acquisition settings for laser intensity, pmt amplification, pinhole aperture (33 μm) and pixel dwell (0.48 μs).

### Immunohistochemistry

Brain-derived neurotrophic factor immunohistochemistry on WT and BDNF^lox/lox^ brain sections were performed on 2% PFA perfused brains cut at 40 μm thick coronal sections, as previously described (Tongiorgi et al., 2004). Slices were developed through Nickel-enhanced diamminobenzidine (DAB) chromogenic reaction for about 20–30 min at room temperature in dark. All passages, except development, were performed in gentle shaking.

### Western Blots

Cells were collected in lysis buffer and the lysates were centrifuged at 10000 rpm for 15 min at 10°C to remove cellular debris. Protein concentration was determined using Bradford assay. Samples were resolved on 12% SDS-PAGE gels, loading 20 μg of PC12 extracts. Proteins were transferred onto nitrocellulose membrane (Schleicher&Schuell) and stained using the different antibodies (**Supplementary Table S3**). Membranes were then washed 3 min × 5 min in 5% non fat milk in PBST and incubated for 1 h at RT with the respective secondary antibody (**Supplementary Table S3**) and membrane developed with chemiluminescent substrate (ECL by GE Healthcare). X-ray films were scanned and quantified by the Quantity One^®^ 4.6.6. software (Bio-Rad, Hercules, CA, USA).

### Colocalization Analysis

Confocal images were acquired by a Nikon Eclipse C1 confocal microscope mounted on a Nikon TE-2000U inverted microscope using a Plan-Apochromat 60x/1.4 oil-immersion objective. Sequential scanning laser was used to avoid cross talk between different fluorochromes. Only pyramidal neurons with clear dendrites were acquired, with a z-stack thickness of 200 nm (12 stacks). At least 21 neurons from three independent experiments were measured for colocalization analysis. Regions of interest (ROI) had a 10 μm × 5 μm × 2.4 μm size located in the proximal (10 μm far from soma) and distal (at least 60 μm far from soma) compartments of apical dendrites. After ROI selection images were cropped, then background was automatically subtracted using Imaris software (Bitplane). Images were then deconvolved using Huygens software with classical CMLE algorithm. “Coloc” function of Imaris was used to perform colocalization analysis Automatic threshold was applied to each image before Manders coefficients calculation. Images of colocalization between mRNA and RBPs are Z-stack maximum projection of representative dendrite shafts analyzed. The images were post-processed to linearly increase brightness equally in the whole panel for visualization purpose.

### Quantitative Imaging Analysis and Statistics of Non-radioactive *In situ* Hybridization

Non-radioactive ISH was analyzed by viewing stained cultures under bright-field illumination as previously described (Tongiorgi et al., 1997; Chiaruttini et al., 2009) with slight modifications. In detail, individual preparations were coded and analyzed in a blind manner with respect to treatments. Bona-fide pyramidal neurons were randomly sampled from each culture and acquired by a Nikon AXM1200 digital camera on a Nikon E800 Microscope with interference contrast-equipped lens (60x magnification) and then analyzed with the image analysis program ImageJ 1.44 (NIH) using the “Straighten” plugin. Dendritic targeting of *BDNF* transcripts in apical dendrites of pyramidal neurons was evaluated as the maximum distance of dendritic labeling (MDDL) measured in μm from the soma using the “Trace” function of the software Image-ProPlus 4.0 (Media Cybernetics). Straightened dendrites were “traced” in a conservative manner, starting from the base of dendrite after soma and up to the point in which *in situ* labeling was clearly distinguishable. At least 150 neurons from four independent experiments were measured for each condition. For both MDDL analysis and semi-quantitative densitometric analysis of BDNF mRNA ISH levels, we used standardized development time of alkaline phosphatase reaction of 40 min at RT. Labeling intensity was measured as a scale of gray levels from 0 = no staining to 255 = saturated black staining, using the software Image-ProPlus 4.0 (Media Cybernetics) as previously described (Chiaruttini et al., 2009). Background level for each dendrite was determined by evaluation of the mean gray value in the distal portion of apical dendrites of transfected neurons in which *in situ* labeling was not present. Background staining was then substracted from each dendrites before averaging. This procedure produces densitometric data that are expressed in arbitrary units (or gray levels). This densitometric method to quantify ISH does not require signal normalization, as previously validated in ours and other laboratories (An et al., 2008; Chiaruttini et al., 2009). Statistical significance among groups was evaluated performing Kruskal–Wallis one-way ANOVA on ranks, followed by a multiple comparison procedure with Dunn’s method.

## Results

### Inducible Dendritic Targeting of Endogenous *BDNF* mRNA

We previously showed that neuronal depolarization and BDNF induce dendritic localization of BDNF mRNA *in vitro* (Tongiorgi et al., 1997; Righi et al., 2000), however, it remained undetermined if other neurotrophins can also induce BDNF mRNA localization in dendrites. To investigate the effect of neurotrophins we treated primary rat hippocampal neurons at 6 days *in vitro* (DIV) for 3 h with NGF, BDNF, NT-3, NT-4 (50 ng/ml) or 10 mM KCl. Endogenous *BDNF* mRNA was detected in isolated cultured cells using non-radioactive ISH using a probe targeting the CDS and hence recognizing all the possible *BDNF* transcripts expressed (**Figure 1A**). Dendritic targeting of *BDNF* transcripts was evaluated as the MDDL from the soma, normalized to untreated condition. At least 150 neurons from four independent experiments were measured for each condition. KCl treatment induced a 2.83 ± 0.17 fold increase of *BDNF* mRNA targeting with respect to basal culturing conditions (**Figures 1B,C**). Stimulation with BDNF and NT-3 induced also a significant fold increase compared to untreated cells (1.59 ± 0.11 and 1.49 ± 0.10, respectively), while NGF and NT-4 did not significantly alter *BDNF* mRNA distribution along dendrites (**Figures 1B,C**). Activity of neurotrophins and KCl solution used in these experiments was demonstrated by translocation of *c-fos* in the nuclei (**Supplementary Figure S1**). Blocking Trk receptors activity with the inhibitor K252a prevented *BDNF* mRNA dendritic translocation after application of BDNF or NT-3 (**Figure 1C**). Inhibition of Trk signaling by K252a also depressed KCl-dependent dendritic targeting, suggesting the contribution of neurotrophin signaling during KCl-induced neuronal depolarization, as previously described for BDNF stimulation (Righi et al., 2000). These data provide strong evidence that distal dendritic localization of endogenous *BDNF* mRNA can be induced in hippocampal neurons by depolarization, BDNF and NT-3.

### Long and Short *BDNF* 3′ UTRs Respond Differently to NT-3 and BDNF

**FIGURE 2:**
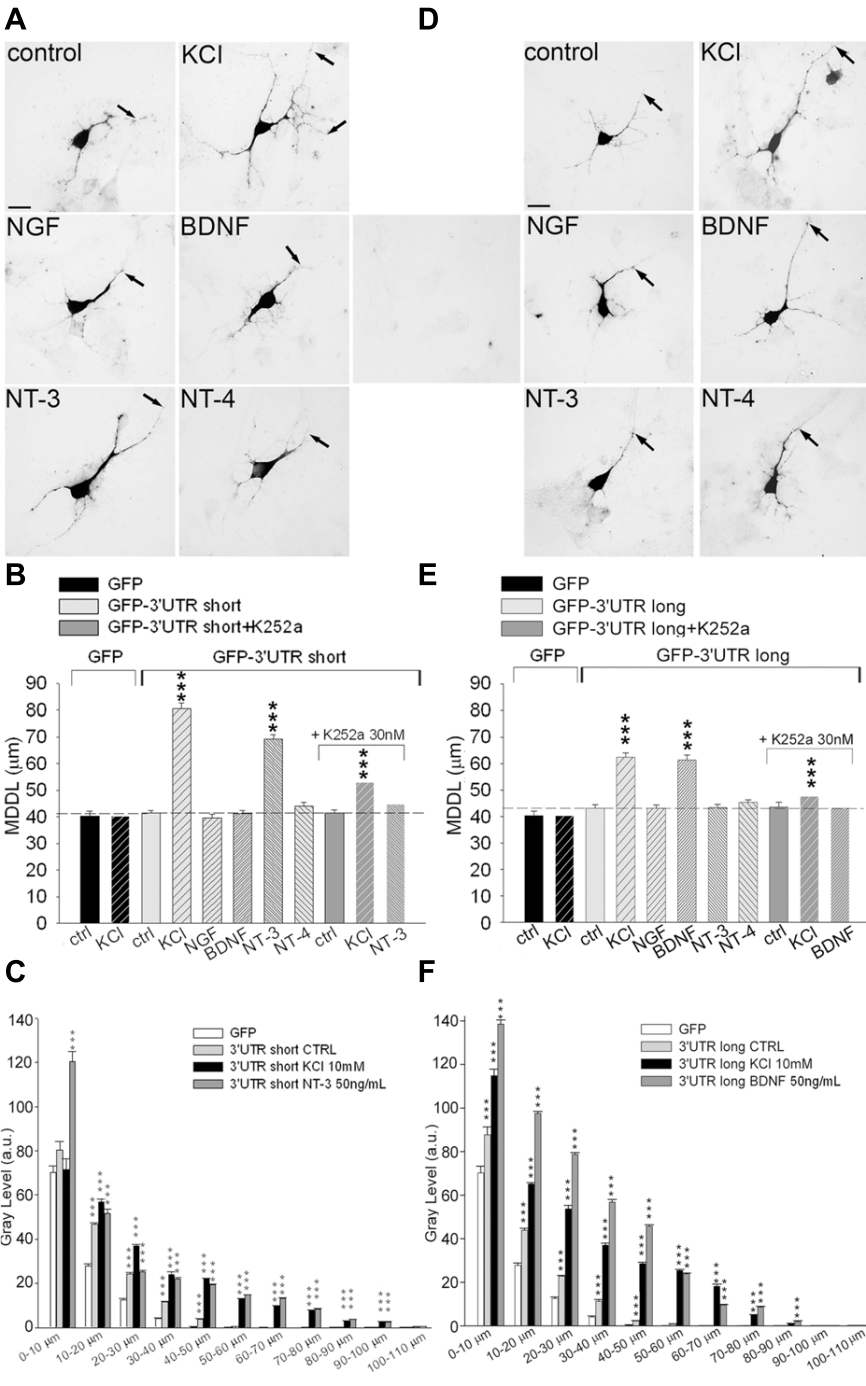
**Inducible targeting of short and long BDNF 3′ UTR chimeric mRNAs.** ISH on hippocampal neurons transfected with either GFP-3′ UTR short **(A)** or long **(D)** in unstimulated (control) and stimulated conditions. **(D)** Central panel, control ISH using GFP sense probe on transfected cells. Arrows indicate MDDL (MDDL); scale bar 10 μm. MDDL quantification of BDNF 3′ UTR short **(B)** or BDNF 3′ UTR long **(E)** following KCl (10 mM) and neurotrophins (50 ng/ml) treatments, alone or in combination with the Trk inhibitor K252a (30 nM). Densitometric analysis of *GFP* mRNA distribution along dendrite length (0–110 μm) in neurons transfected with GFP-3′ UTR short **(C)** or long **(F)**, in resting conditions of after treatment with KCl, NT-3 or BDNF (^∗∗∗^*P* < 0.001 vs. control, Kruskal–Wallis one way ANOVA on Ranks followed by Dunn’s multiple comparison test).

We previously showed that upon treatment with high KCl, the short 3′ UTR BDNF can target the mRNA of a reporter gene to the distal dendrites of cultured neurons (Baj et al., 2011). To investigate the role of short and long 3′ UTRs with respect to the stimulation with activity, BDNF or NT-3, primary rat hippocampal neurons were transfected with GFP-3′ UTR short or GFP-3′ UTR long chimeras. Cultures were stimulated for 3 h with 10 mM KCl or 50 ng/ml of each neurotrophin, and neurons were subjected to non-radioactive ISH using a DIG-labeled GFP antisense probe and finally, the MDDL from the soma was measured (**Figure 2**). No unspecific staining was detected using GFP sense strand probes (**Figure 2D**, middle panel). In resting neurons, the short 3′ UTR was mainly confined in the proximal dendritic compartment (MDDL = 41.56 ± 0.82 μm) similar to GFP mRNA (MDDL = 40.34 ± 1.72 μm; **Figures 2A,B**). GFP mRNA localization was not modified by neuronal depolarization (MDDL = 40.36 ± 1.87 μm), whilst GFP-3′ UTR short mRNAs showed a significant translocation in distal dendrites both after high potassium depolarization (MDDL = 80.57 ± 1.94 μm, *P* < 0.001) or treatment with NT-3 (MDDL = 69.14 ± 1.61 μm, *P* < 0.001). NGF, BDNF or NT-4 had no effect on GFP-3′ UTR short mRNA localization (**Figures 2A,B**). Trk tyrosine kinase inhibitor K252a abolished the GFP-3′ UTR short dendritic localization in response to NT-3 application, in accordance with the results previously seen for endogenous BDNF mRNA (**Figure 2B**). Similarly, KCl-induced translocation was significantly impaired, suggesting that a NT3-dependent mechanism plays a substantial role in activity-induced dendritic targeting of short 3′ UTR transcripts (**Figure 2B**).

Localization of the GFP-3′ UTR long chimera did not differ significantly from the GFP reporter alone in unstimulated cultures (MDDL = 43.14 ± 1.36 μm). However, depolarization induced strong accumulation of this mRNA in distal dendrites (MDDL = 62.44 ± 1.44 μm, *P* < 0.001), similarly to the short 3′ UTR constructs (**Figures 2D,E**). Furthermore, application of 50 ng/ml BDNF (but not NT-3, NT-4 nor NGF) induced dendritic translocation of the GFP-3′ UTR long at distances comparable those induced by KCl depolarization (MDDL = 61.38 ± 1.92 μm). K252a application abolished BDNF-induced, and significantly reduced KCl-dependent, 3′ UTR long mRNA distal targeting, suggesting that BDNF signaling is required to enhance the depolarization effect on mRNA localization (**Figure 2E**). The ISH signals from the very same neurons were quantified by densitometric analysis throughout the entire dendritic length, leading to results comparable to those obtained with MDDL measurements (**Figures 2C,F**). Taken together, these results provide clear evidence that both the long and short 3′ UTR sequences are involved in inducible dendritic localization of *BDNF* mRNA. Dendritic targeting of *BDNF* mRNA is modulated by neuronal depolarization (KCl) for both regions but is specifically regulated by NT-3 for the short 3′ UTR and by BDNF for the long 3′ UTR.

### Activity-dependent Dendritic Targeting of *BDNF* Short 3′ UTR mRNA *In vivo*

**FIGURE 3:**
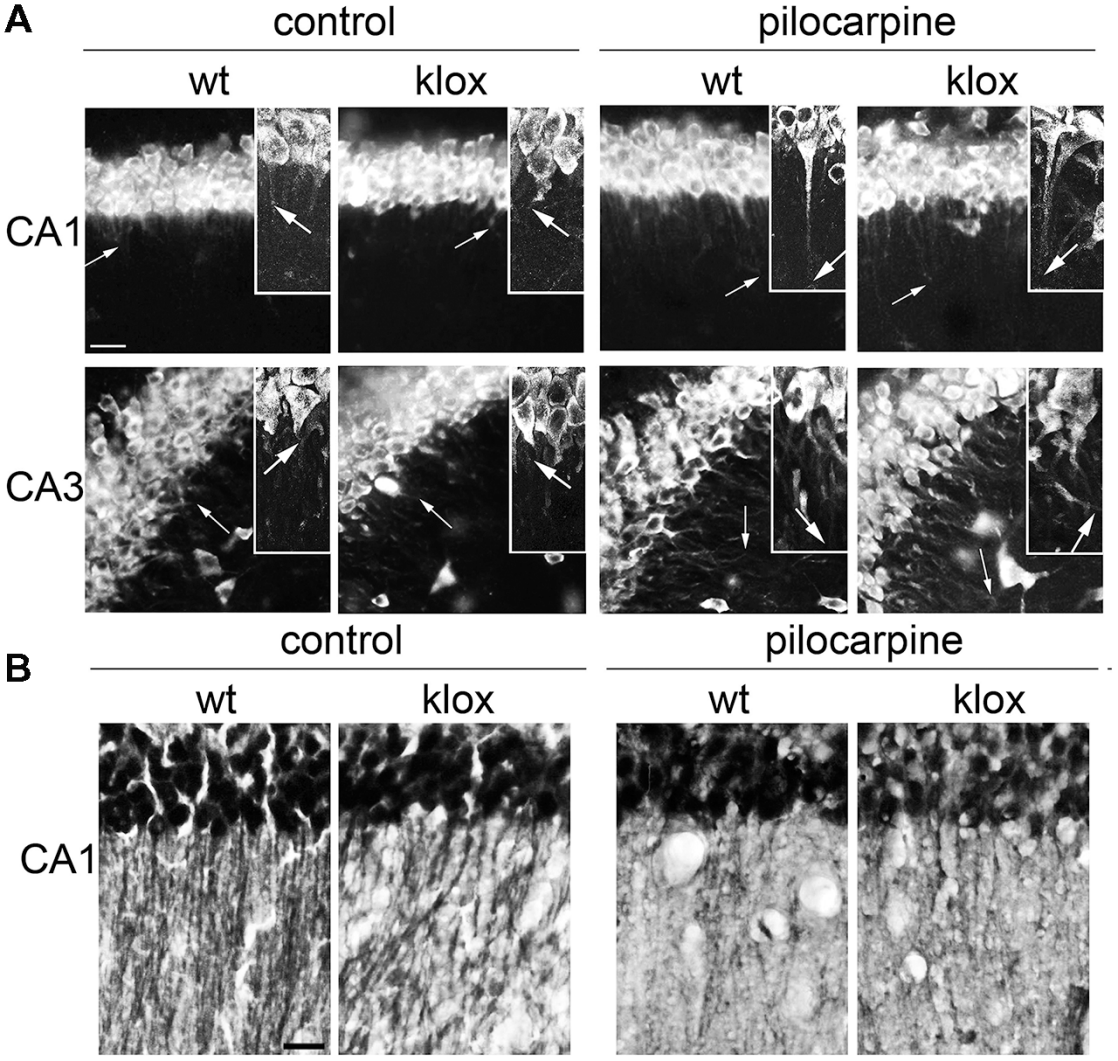
**Endogenous BDNF 3′ UTR short localization *in vivo*.** **(A)** Representative images of fluorescent *in situ* hybridization (FISH) of endogenous BDNF CDS in CA1 (upper panel) and CA3 (bottom panel) in wild type (WT) and BDNF^lox/lox^ transgenic mouse devoid of the long BDNF 3′ UTR sequence (lox) in basal conditions (left) and upon 3 h of pilocarpine-induced seizures (300 mg/kg, 3 h; right). Arrows indicate the maximal distance of dendritic labeling. **(B)** Representative images of BDNF protein immunostaining in CA1 area from the same brains used for FISH **(A)**; scale bar, 20 μm for **(A)** and 40 μm for **(B)**.

A previous study suggested that BDNF transcripts of BDNF^lox/lox^ mice, lacking a functional long 3′ UTR, are confined in the soma *in vivo* (An et al., 2008). However, activity-dependent targeting *in vivo* of short 3′ UTR transcript was not evaluated. To determine whether the short 3′ UTR form is targeted to dendrites also *in vivo*, we took advantage of the same BDNF^lox/lox^ mouse model (Gorski et al., 2003). In WT mice in resting conditions, *BDNF* mRNA was detected in the soma and in the proximal dendrites of CA1 and CA3 neurons while in BDNF^lox/lox^ mice it was detectable only in the soma, as previously described (An et al., 2008; **Figure 3A**). However, after treatment with the pro-epileptic drug pilocarpine for 3 h, localization of *BDNF* transcripts in dendrites was observed both in WT and BDNF^lox/lox^ hippocampi, confirming the finding that *BDNF* mRNA splice variants with a short 3′ UTR can be transported in dendrites after stimulation (**Figure 3A**). Interestingly, in BDNF^lox/lox^ neurons a punctate distribution of BDNF mRNA was observed, whilst a more uniform signal was seen in WT animals. These differences may be due to the fact that the long 3′ UTR is lacking from distal compartments of BDNF^lox/lox^ neurons and therefore staining for *BDNF* mRNA is generally weaker. BDNF^lox/lox^ mice display decreased BDNF protein dendritic levels despite the total amount of protein is comparable (An et al., 2008). To test if pilocarpine treatment is able to increase BDNF protein levels in BDNF^lox/lox^ mice, brain sections from WT and mutated animals were immunostained with an anti-BDNF antibody. Hippocampal neurons of pilocarpine-treated WT and BDNF^lox/lox^ mice were found to display comparable labeling in distal dendrites (**Figure 3B**), providing further evidence that BDNF can reach distal dendrites even if it is translated from transcripts expressing the 3′ UTR short only.

### Multiple RNA Binding Proteins are Associated to *BDNF* mRNA

**FIGURE 4:**
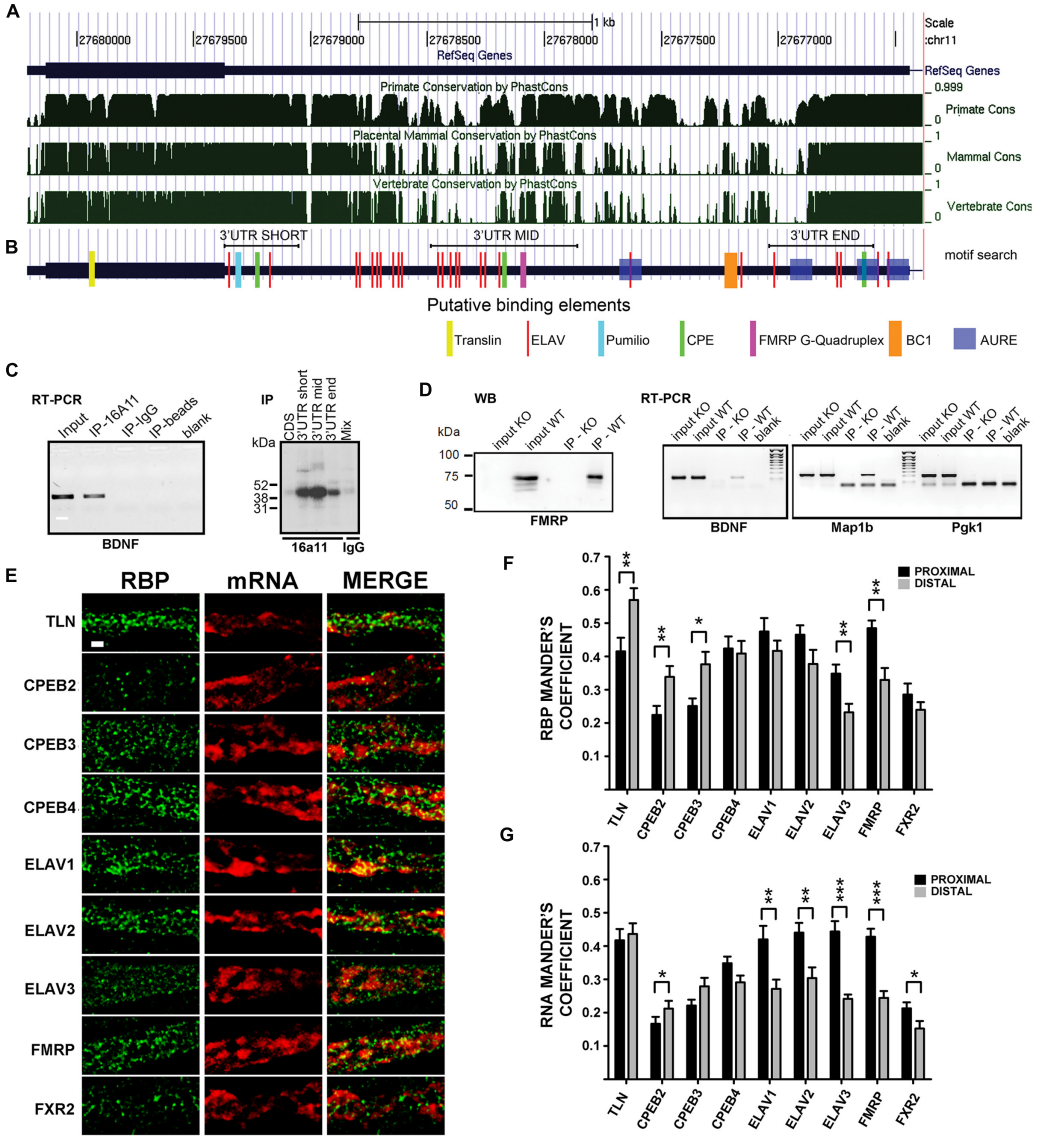
**Brain-derived neurotrophic factor mRNA interaction with RNA binding proteins (RBPs).** **(A)** Graphical view of homology of BDNF coding regions and 3′ UTR among Primates, Mammals, and vertebrates. Similarity scores range from 0 (=no homology) to 1 (identical sequence). Numbers indicate the nucleotide position of BDNF gene on chromosome 11. **(B)** Putative binding sites for RBPs indicated with different colors at the corresponding positions along *BDNF* mRNA. Positions of the short 3′ UTR, long-mid 3′ UTR and long-end sequences used in UV-CLIP assays in **(C)** are also shown. (**C**, left panel) Reverse transcriptase-PCR of BDNF mRNA immunoprecipitated with an antibody against the neuronal ELAV proteins (IP-16A11), or a control IgG antibody (IP-IgG) or no antibody (IP-beads). Total brain RNA (Input) and no sample (Blank) were used as a positive and a negative control, respectively; (right panel) UV-CLIP assay with an anti-neuronal ELAV proteins antibody (IP-16a11) to immunoprecipitate RNA-protein complexes formed in presence of murine brain protein lysate and radiolabeled BDNF CDS, 3′ UTR short, or 3′ UTR long-mid and 3′ UTR long-end ribobrobes. As a negative control, UV-CLIP was performed using an anti-IgG antibody with a mix of all the *BDNF* riboprobes used. **(D)** Immunoblot assay to test specificity of the anti-FMRP antibody in inputs and immunoprecipitates from WT and Fmr1 knockout (KO) mice (left panel). Reverse transcriptase-PCR of *BDNF* mRNA recovered from immunoprecipitates from WT and *Fmr1* knockout lysates upon CLIP assay (middle panel). *Map1b*, a known mRNA target of FMRP protein, was used as a positive control, while *Pgk1*, an unrelated mRNA, as a negative control. Blank, no sample in Reverse transcriptase-PCR reaction. **(E)** Colocalization analysis of endogenous *BDNF* mRNA with different RBPs. Immunofluorescence signal from the different RBPs (green) and from endogenous *BDNF* mRNA (red) in proximal dendrites are shown separately (RBP, mRNA) and merged (MERGE). Images are Z-stack maximum projection of 12 stacks confocal images. Scale bar: 1 μm. **(F,G)** Graphs report the Manders coefficient (Y-axis) of RBPs signal colocalized with endogenous *BDNF* mRNA **(F)** and *BDNF* mRNA signal colocalized with the different RBP **(G)** in proximal (dark gray) and distal (light gray) dendrites. Data are reported as mean ± SEM. Statistical significance of proximal vs. distal Manders coefficients of colocalization was evaluated performing *t*-test if the normality test was passed, or Mann–Whitney Rank-Sum test (^∗^*P* < 0.05; ^∗∗^*P* < 0.01; ^∗∗∗^*P* < 0.001).

Previous evidence showed that *BDNF* mRNA trafficking and stabilization requires interaction with RBPs (Chiaruttini et al., 2009; Oe and Yoneda, 2010; Louhivuori et al., 2011). To identify the RBPs interacting with the 3′ UTR short or long, we used an *in silico* approach to detect evolutionary conserved regions and putative binding proteins sites on each 3′ UTR variant. BDNF sequences were compared among primates, mammals, and vertebrates (see **Supplementary Figure S2**) using phast Cons alignment software (Siepel et al., 2005). Several regions were found to be highly conserved (**Figure 4A**), with the short 3′ UTR region being the most conserved one, followed by the mid and terminal regions of the long 3′ UTR. To recognize binding motifs for RBPs, the bioinformatic analysis was focused on regions highly conserved among vertebrate species, which are more likely to share an evolutionary conserved biological function. We searched for consensus recognition sequences for some RBP families including cytoplasmatic polyadenylation element binding proteins (CPEBs), embryonic lethal abnormal vision like proteins (ELAVs) and Fragile X mental retardation protein (FMRP), which are known to bind to short, well-defined RNA sequences and are involved in RNA transport, translation, and stabilization. The short 3′ UTR (nts 1–321) displays one CPEB binding site and two different conserved sites for ELAV proteins (the upper 5′ site is referred here as to “ELAV up” and the 3′ most as “ELAV down”; **Figure 4B**). Additionally, in this region we found two other conserved sites, a Nanos response element (NRE) and a CUG rich region representing a potential target for Pumilio, a translational regulator (Wickens et al., 2002), and for the CUG-binding protein (CUGBP), known to regulate splicing, deadenylation, mRNA stability and translation (Philips et al., 1998; Timchenko et al., 1999; Ladd et al., 2001; Savkur et al., 2001; Paillard et al., 2003; Moraes et al., 2006; Mori et al., 2008). Despite the high conservation degree of these sequences, we did not consider Pumilio and CUGBPs in the present study, since so far there are no clear reports assessing their involvement in regulation of mRNA transport. Even if the BDNF long 3′ UTR showed a higher degree of variability among species when compared to the short 3′ UTR, three conserved “hot spots” were identified (**Figures 4A,B**). The first conserved region, located at the boundary with the 3′ UTR short, presents an “ELAV rich region” with a cluster of binding sites for the ELAV-1 family member (HuR, red bars in **Figure 4B**). A nearby second conserved sequence, in the center of the long 3′ UTR, is characterized by a G-quartet like structure, known to be recognized by FMRP (Darnell et al., 2001). Finally, a third region located at the end of the long 3′ UTR shows a pattern of *cis*-elements similar to the one found in the short 3′ UTR, with CPEs and ELAV recognition sites. Additionally, we found one BC1 (Rozhdestvensky et al., 2001) and several AU-rich response element (AURE), especially in the 3′ terminal region of the long 3′ UTR, which support a previously suggested possible involvement of hnRNPA2 RBP (Raju et al., 2011).

In order to detect an interaction of ELAVs with *BDNF* transcripts, we performed a RNA immunoprecipitation (RIP) assay on mouse brain lysates using a pan-neuronal ELAV (nELAV) antibody, followed by Reverse transcriptase-PCR. This analysis showed that *BDNF* mRNA was selectively recovered from anti-nELAV immunoprecipitates (IPs), but not from control-IgG IPs or in presence of beads alone (**Figure 4C**, left panel). To test the specificity of binding of nELAVs to the BDNF 3′ UTR sequence, we used radiolabeled riboprobes corresponding to BDNF short 3′ UTR and CDS in UV Cross-Linking Immunoprecipitation (UV-CLIP) assays with mouse brain protein lysates. In addition, we used two riboprobes corresponding to the central (mid, nts 890–1510) or terminal (end, nts 2339–2790) regions of the 3′ UTR long sequence that also contained ELAV consensus binding motifs (**Figures 4B,C**, right panel). No RNA-protein complex formed in presence of the CDS-specific riboprobe, whilst specific nELAV-*BDNF* RNA complexes were recovered in presence the short, mid and end 3′ UTR riboprobes (**Figure 4C**, right panel). To test for FMRP binding, we performed similar CLIP experiments with mouse brain lysates using a previously described polyclonal rabbit anti-FMRP antibody (Davidovic et al., 2011). The specificity of the anti-FMRP antibody used was first proven by immunoprecipitation assays using WT and *FMR1* knockout mouse brains as a negative control (**Figure 4D**, left). Accordingly, endogenous *BDNF* mRNA could be amplified via Reverse transcriptase-PCR from WT but not *FMR1* knockout mouse brains immunoprecipitates (**Figure 4D**, middle panel). *Map1b* and *Pgk1* mRNAs were used as positive and negative controls for FMRP binding, respectively (**Figure 4D**, right panel). Previous studies already demonstrated that CPEBs physically bind to BDNF CDS and 3′ UTR mRNA (Oe and Yoneda, 2010) and therefore the binding experiments for these proteins were not performed.

To verify if the above mentioned RBP are colocalized with BDNF mRNA in dendrites, we performed double labeling experiments with FISH against endogenous *BDNF* transcripts coupled to immunofluorescence (IF) for RBPs family members in unstimulated cultures (**Figure 4E**). Tyramide Amplification System was employed to visualize endogenous *BDNF* mRNA in distal dendrites, due to its intrinsic low abundance in such compartment of resting cultured neurons (Baj et al., 2013; Will et al., 2013). The different RBPs taken in account in this study showed a similar distribution in dendrites, suggesting that they represent shared components of dendritic mRNA transporting granules. In proximal dendrites (**Figures 4E–G**), in unstimulated cultures, almost half of the staining spots of Translin, CPEB4, ELAV1, ELAV2, and FMRP were colocalized with endogenous *BDNF* mRNA (Manders coefficient 0.415 ± 0.041, 0,424 ± 0.036, 0.475 ± 0.040, 0.465 ± 0.028, and 0.485 ± 0.023, respectively), while other transporting granules were less colocalized, with Manders coefficient values ranging from 0.224 to 0.348. Similarly BDNF mRNA signal results to be more colocalized in the proximal compartment with Translin, ELAV1, ELAV2, ELAV3, and FMRP granules (Manders coefficient 0.417 ± 0.034, 0,420 ± 0.041, 0.440 ± 0.029, 0.444 ± 0.031, and 0.423 ± 0.024, respectively). In distal compartments more than half of the transporting granules stained for Translin were colocalized with *BDNF* mRNA (Manders coefficient 0.579 ± 0.035), while other RBPs display similar colocalization degree with RNA, except for the less colocalized ELAV3 and FXR2 (Manders coefficient 0.232 ± 0.026 and 0.240 ± 0.023, respectively). Moreover, *BDNF* mRNA displayed the highest colocalization rate with Translin protein (Manders coefficient 0.436 ± 0.032) confirming its involvement in this mRNA trafficking as previously demonstrated (Chiaruttini et al., 2009) while it showed a heterogeneous colocalization with other RBPs (Manders coefficient ranging from 0.152 to 0.304). CPEB-1 and ELAV-4 were undetectable after FISH, therefore they have been omitted in the colocalization analysis. Statistical comparison revealed that RBPs colocalization with BDNF mRNA was significantly higher in distal dendrites than in proximal dendrites for translin, CPEB2, CPEB3, while it was higher in proximal than in distal for ELAV3 and FMRP, and was equally distributed for CPEB4, ELAV1 and 2, and FXR2 (**Figure 4F**). Viceversa, when colocalization was analyzed taking BDNF mRNA as reference, we found that BDNF mRNA was significantly more localized with ELAV1, 2, 3, FMRP and FXR2 in proximal dendrites than in distal (**Figure 4G**), suggesting that this mRNA and these RBPs interact more in the initial segment of the dendrites.

### Different Set of RBPs Regulates Long and Short 3′ UTRs Dendritic Targeting

**FIGURE 5:**
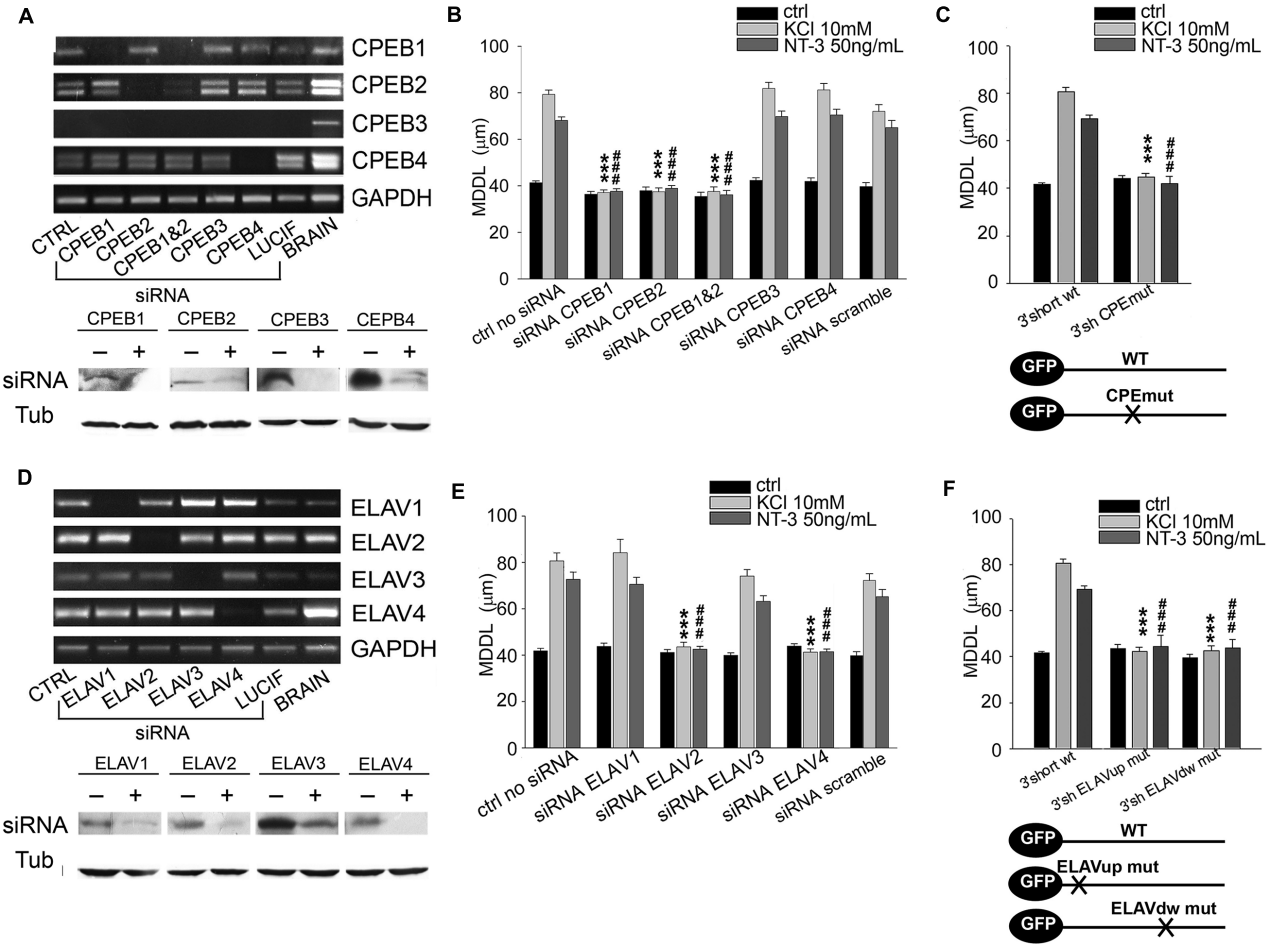
**Role of CPEB and ELAV proteins in BDNF 3′ UTR short activity-dependent targeting.** **(A,D)** Representative semiquantitative Reverse transcriptase-PCR for CPEBs (**A**, upper panel) and ELAVs (**D**, upper panel) expression levels in control neurons (CTRL), and in neurons silenced with siRNA specific for the indicated RBP or with a scramble siRNA (LUCIF) in comparison to those of total rat brain lysates, taken as positive control. GADPH expression level has been reported for normalization. Representative western blots on lysates from control (-) or silenced (+) PC12 cells with the CPEB (**A**, bottom panel) and ELAVs (**D**, bottom panel) -specific siRNAs. Tubulin was blotted for normalization. **(B)** MDDL quantification of ISH for GFP sequence on neurons transfected with GFP-3′ UTR short construct upon specific silencing of CPEB family members (1, 2, 3, 4) in presence of KCl (10 mM) or NT-3 (50 ng/ml). Controls are represented either by cells not treated with siRNAs or treated with a scramble siRNA (upper panel). **(C)** MDDL quantification of ISH for GFP sequence on neurons transfected with GFP-3′ UTR short construct WT and mutated in the CPEB binding site (upper panel). Representation of transcripts used for the experiments (bottom panel). **(E)** MDDL quantification of ISH for BDNF 3′ UTR short upon silencing of ELAV family members (1, 2, 3, 4) in presence of KCL (10 mM) or NT-3 (50 ng/ml) stimuli. Controls are represented either by cells not treated with siRNAs or treated with a scramble siRNA (upper and bottom panel). **(F)** MDDL quantification of ISH for BDNF 3′ UTR short WT or with mutated ELAV binding sites (upper panel). Representation of transcripts used for the experiments (bottom panel; ANOVA all vs. control with same treatment, ^∗∗∗^*P* < 0.001; ###*P* < 0.001).

**FIGURE 6:**
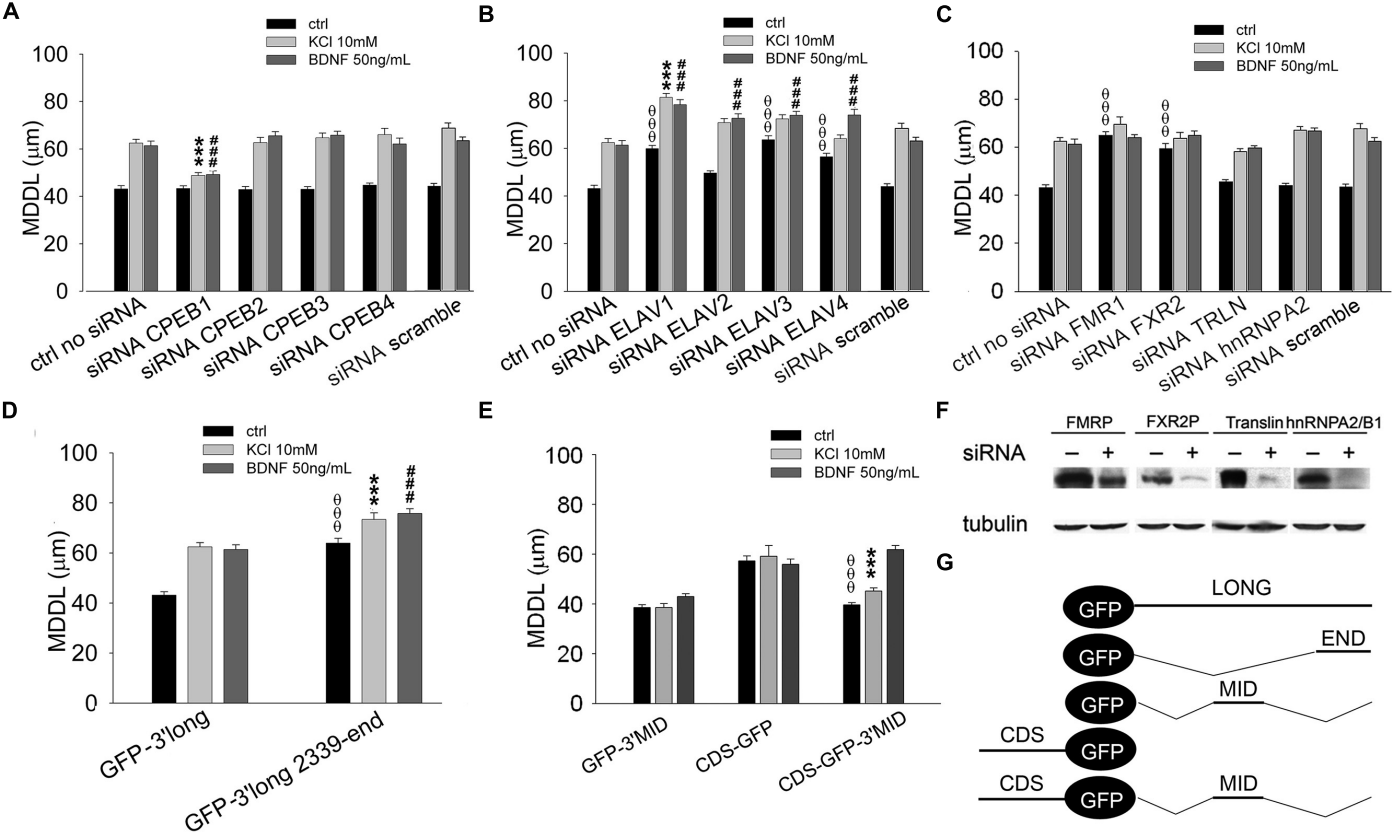
**Role of CPEBs, ELAVs, and FMRP proteins in *BDNF* 3′ UTR long activity-dependent targeting.** **(A–C)** MDDL quantification of ISH for GFP sequence on neurons transfected with GFP-3′ UTR long construct upon silencing of different RBPs (CPEB-1-4, **A**; ELAV-1-4, **B**; FMR1, FXR2, TRLN, hnRNPA2, **C**) in resting conditions or after KCl (10 mM) or BDNF (50 ng/ml) treatment. Controls are represented either by cells not treated with siRNAs or treated with a scramble siRNA (upper panel). **(D)** MDDL quantification of ISH for GFP sequence on neurons transfected with GFP-3′ UTR long construct containing full-length or final portion (nt 2339-end) of *BDNF* 3′ UTR long in resting conditions or after KCl (10 mM) or BDNF (50 ng/ml) treatment. **(E)** MDDL quantification of ISH for GFP sequence on neurons transfected with BDNF CDS-GFP and BDNF CDS-3′ UTR MID-GFP constructs, in resting conditions and after KCl (10 mM) or BDNF (50 ng/ml) treatment (left panel). Graphical representation of the constructs used for the experiments is reported in the bottom right panel (ANOVA all vs. control with same treatment, ^θθθ^*P* < 0.001; ^∗∗∗^*P* < 0.001; ###*P* < 0.001). **(F)** Representative western blots on lysates from control (-) or silenced (+) PC12 cells with FMRP, FXR2, Translin, hnRNPA2/B1 specific siRNAs. Tubulin was blotted for normalization. **(G)** Schematic representation of GFP chimaeric transcripts.

To test if the identified RBPs contribute to the inducible mechanism of *BDNF* mRNA targeting to dendrites, dendritic localization of GFP-BDNF 3′ UTRs was measured following KCl, NT-3 or BDNF stimulation in neurons co-transfected with siRNA cocktails against each member of the CPEB, ELAV, or FRXP families. Analysis of siRNA efficacy showed a high degree of silencing of target RBPs (**Figures 5A,D** and **6F**; **Supplementary Table S4**). The specificity of siRNAs used and the lack of off-target effects among homologous members of the CPEB and ELAV families were confirmed by Reverse transcriptase-PCR (**Figures 5A,D**; **Supplementary Table S4**). When considering the short BDNF 3′ UTR, both activity-dependent and NT-3 mediated localization of the reporter was found to require CPEB-1 and CPEB-2, but not CPEB-3 or CPEB-4 (CPEB-1 siRNA KCl MDDL = 37.16 ± 1.12 μm, CPEB-2 siRNA KCl MDDL = 37.48 ± 1.63 μm, ^∗∗∗∗^*P* < 0.001 respect to KCl no siRNA; CPEB-1 siRNA NT-3MDDL = 37.69 ± 1.09 μm, CPEB-2 siRNA NT-3 MDDL = 38.96 ± 1.18 μm, ^###^*P* < 0.001 respect to NT-3 with no siRNA). A control siRNA against luciferase had no effect on *BDNF* mRNA targeting (**Figure 5B**, scramble). Disruption of the CPE binding site through site directed mutagenesis (UUUUAU to CGAUCG, **Figure 5C**) completely suppressed mRNA sorting in dendrites in response to extracellular stimuli, and further confirmed the role of CPEBs in *BDNF* mRNA trafficking (Huang et al., 2006). However, it is unclear whether CPEB 2 can bind directly to the mRNA, since UUUUAU is the consensus sequence for CPEB-1, and a specific CPEB-2 recognition site has not been described yet (Huang et al., 2006). Both ELAV-2 and ELAV-4 were required for KCl and NT-3-dependent transport of the 3′ UTR short (ELAV-2 siRNA KCl MDDL = 43.57 ± 1.99 μm, ELAV-4 siRNA NT-3 MDDL = 41.32 ± 1.39 μm, ^∗∗∗^*P* < 0.001 respect to no siRNA KCl; ELAV-2 siRNA NT-3 MDDL = 42.53 ± 1.28 μm, ELAV-4 siRNA NT-3 MDDL = 41.48 ± 1.14 μm, ^∗∗∗^*P* < 0.001 respect to no siRNANT-3; **Figures 5E,F**). The other two family members, ELAV-1 and -3, did not result involved in dendritic localization of BDNF 3′ UTR short. Involvement of ELAV-2 and-4 RBPs was further confirmed by the fact that disruption of either the 5′ or 3′ ELAV binding motifs (AUUUAU to AGCGCG) blocked BDNF 3′ UTR short dendritic targeting upon stimuli (**Figure 5F**). The relative RNA levels of these mutants were compared to the parental GFP and WT GFP 3′ UTR short mRNAs to rule out the possibility that the lack of dendritic localization of the 3′ UTR mutants could be related to a decreased stability or expression and indeed we did not observe any significant difference (**Supplementary Figures S3A,B**). In summary, these results clearly demonstrate that KCl and NT-3 are able to induce distal dendritic localization of BDNF 3′ UTR short through a complex post-transcriptional regulatory mechanism that requires the interaction of CPEB-1, CPEB-2, ELAV-2, and ELAV-4 RBPs.

A similar approach was used to identify key RBPs modulating the long BDNF 3′ UTR localization in distal dendrites upon KCl or BDNF stimuli. Dendritic targeting of the long isoform required CPEB-1 but not CPEB-2, CPEB-3, or CPEB-4 in both conditions (KCl MDDL = 48.84 ± 1.15 μm, ^∗∗∗^*P* < 0.001 respect to no siRNA KCl, BDNF MDDL = 49.17 ± 1.55 μm, ^###^*P* < 0.001 respect to no siRNA BDNF; **Figure 6A**). Silencing of individual ELAV family members induced an unexpectedly complex pattern of responses. ELAV-1, -3, and -4 down-regulation promoted a significant increase in 3′ UTR long targeting after stimulation and even at resting conditions, in particular following silencing of ELAV-1 (ctrl MDDL = 59.90 ± 1.31 μm, ^θθθ^*P* < 0.001 respect to ctrl no siRNA; KCl MDDL = 81.40 ± 1.64 μm, ^∗∗∗^*P* < 0.001 respect to no siRNA KCl; BDNF MDDL = 78.36 ± 2.13 μm, ^###^*P* < 0.001 respect to no siRNA BDNF; **Figure 6B**). A similar increase in dendritic targeting of the 3′ UTR long was found at resting conditions also with siRNAs against the Fragile-X proteins (FXRPs) FMRP1 and FXR2, consistent with our recent study on total *BDNF* mRNA localization in *Fmr1^-/-^* mice (Louhivuori et al., 2011; ^θθθ^*P* < 0.001, **Figure 6C**). Interestingly, activity- or neurotrophin-induced dendritic targeting was normal in absence of FMRP or FXR2 (**Figure 6C**). Additionally, the role of other two RBPs, Translin and hnRNPA2/B1 was tested. Translin was used as an internal control since this protein does not appear to be involved in the sorting of 3′ UTR long while hnRNPA2/B was tested since putative AURE recognition elements have been identified in the terminal part of the 3′ UTR long. No significant alterations of BDNF 3′ UTR long mRNA targeting were observed after Translin or hnRNPA2/B1 silencing (**Figure 6C**). These findings suggest that a specific set of RBPs is able to retain 3′ UTR long-containing splice variants in the proximal dendritic compartment of resting neurons while other RBPs are involved in the dendritic targeting. These “soma-restrictive” proteins display clustered recognition sites in the 3′ UTR long central region which is remarkably well-conserved throughout the evolution (nts 890–1510, **Figure 4B**) while RBPs involved in the dendritic targeting appeared located mostly at the 3′ end of long 3′ UTR region (nts 2339–2790, **Figure 4B**).

### The Long 3′ UTR Contains Two Distinct RNA Localization Signals with Opposing Functions

We speculated that the central region of the 3′ UTR long might contain a retention signal, able to counteract both the constitutive dendritic targeting signal in the coding region of *BDNF* mRNA (Chiaruttini et al., 2009) and the inducible signal present in the terminal part of the 3′ UTR long. To test this hypothesis, the terminal 3′ UTR long sequence (nts 2339–2790) was inserted at the 3′ of the GFP reporter (GFP-3′ UTR END, **Figure 6G**). This chimera displayed an increased distal targeting with respect to the full length 3′ UTR long under basal conditions (MDDL = 63.96 ± 1.93 μm, ^OOO^*P* < 0.001 respect to ctrl 3′ UTR long), comparable to that observed when ELAV-1, -3, or -4 were silenced (compare **Figures 6D** and **6B**). At the same time, both KCl and BDNF induced a small but significant increase in distal transport of the terminal 3′ UTR long sequence with respect to the full-length 3′ UTR long (3′ UTR END KCl MDDL = 73.41 ± 2.61 μm, ^∗∗∗^*P* < 0.001 respect to KCl 3′ UTR long; 3′ UTR END BDNF MDDL = 75.79 ± 1.88 μm, ^###^*P* < 0.001 respect to BDNF 3′ UTR long, **Figure 6D**). To test the role of the central region of the 3′ UTR long, the sequence 849–1533 nt was cloned downstream the GFP reporter (GFP-3′ UTR MID, **Figure 6G**). The 3′ UTR MID fragment did not respond to any stimulus suggesting the absence of DTE while the CDS fragment was constitutively localized in dendrites (**Figure 6E**). However, when the central region was inserted at the 3′ end of the CDS-GFP construct (CDS-GFP-3’MID, **Figure 6G**; Chiaruttini et al., 2009), it completely suppressed the constitutive dendritic localization of the CDS (CDS-GFP-MID ctrl MDDL = 39.65 ± 0.91 μm vs. CDS-GFP ctrl MDDL = 57.25 ± 2.0 μm, ^θθθ^*P* < 0.001, **Figure 6E**). Notably, this retention signal was relieved by BDNF (CDS-GFP-MID BDNF MDDL = 61.85 ± 1.63 μm), but not by stimulation with KCl (CDS-GFP-MID KCl MDDL = 45.17 ± 1.28 μm, ^∗∗∗^*P* < 0.001 respect to KCl GFP-CDS, **Figure 6E**).

## Discussion

This study provides evidence of dendritic transport of both short and long *BDNF* 3′ UTR variants and unravels the core molecular mechanisms underlying inducible dendritic targeting of these two *BDNF* mRNA forms. A remarkable outcome of this study is the strikingly selective response to NT-3 for short 3′ UTR and to BDNF for long 3′ UTR. Activity- and NT3-dependent dendritic targeting of 3′ UTR short requires CPEB-1,2 and ELAV-2,4 while dendritic targeting of long 3′ UTR relies on a more complex mechanism. This includes BDNF-dependent release of soma-retention signals mediated by ELAV-1,3,4, FMRP and FXRP2, and KCl/BDNF-activation of inducible targeting signals mediated by CPEB-1.

We found that Neurotrophin-3 activates dendritic targeting of *BDNF* short 3′ UTR and BDNF activates the long 3′ UTR form. NT-3 was previously shown to be able to redistribute mRNAs to distal dendrites in cultured neurons but the mechanism has remained unknown (Knowles and Kosik, 1997). In addition, NT-3 is known to promote beta-actin mRNA localization within axonal growth cones through a mechanisms requiring blockade of microtubule depolymerisation following activation of cAMP-dependent protein kinase A (PKA; Zhang et al., 1999). A remarkable finding of our study is that NT-3 can regulate the trafficking selected isoforms of BDNF. The reciprocal interplay between BDNF and NT-3 has been previously investigated *in vitro* and *in vivo* (Lindholm et al., 1994; McAllister et al., 1997; Patz and Wahle, 2006). BDNF and NT-3 have been shown to play counteracting effects in regulating cortical dendritic growth during development (McAllister et al., 1997). Interestingly, the receptor for NT-3, TrkC, is the earliest neurotrophin receptor expressed during brain development, followed by a later expression of TrkB (Bernd, 2008). Moreover, in visual cortex NT-3 mRNA levels, detected prenatally, dramatically decrease at later stages of development, with an opposing trend for BDNF mRNA (Lein et al., 2000; Patz and Wahle, 2006). BDNF and NT-3, which act presumably by activating TrkB and TrkC receptors, coupled to activation of similar signaling mechanisms, have differential effects on dendritic targeting of BDNF mRNAs. Previous studies suggest, however, that mRNA dendritic transport activated by NT-3 or BDNF may be induced through different mechanisms which also govern protein translation at synapses (Leal et al., 2014). For instance, while it was previously shown that BDNF promotes the transport of its own mRNA along dendrites through a PI3K-dependent mechanism (Righi et al., 2000), NT-3 was shown to induce dendritic trafficking of beta-actin mRNA through a PKA-dependent mechanism (Zhang et al., 1999).

There is a general consensus that BDNF mRNA levels in distal dendritic processes are low both under resting conditions and after stimulation *in vitro* and *in vivo* (Tongiorgi et al., 1997, 2004; An et al., 2008; Will et al., 2013). It has been proposed that *BDNF* mRNA is present in low amounts in dendrites, most likely because of a short half-life compared to other dendritic mRNAs (Will et al., 2013) and it has been suggested that neuronal activity may selectively increase stability of the long 3′ UTR *BDNF* mRNA by interactions with the RBP ELAV-1/HuD (Fukuchi and Tsuda, 2010; Allen et al., 2013). However, a previous study showed that *BDNF* short and long 3′ UTR variants are equally stabilized by activity (Fukuchi and Tsuda, 2010) and interactions of ELAV-1/HuD with the short 3′ UTR were described (Lim and Alkon, 2012). The lack of effect of KCl depolarization on *BDNF* mRNA decay in our conditions, suggests that its increase in dendrites is due to active transport rather than increased stability. Collectively, these previous studies and the results presented here, suggest that the most likely cause of the low levels of *BDNF* mRNA observed in distal dendrites is the result of a tight activity-dependent regulation of transport, which limits the quantity of *BDNF* mRNA available in dendrites.

Neither short nor long BDNF 3′ UTR mRNAs were found to require CPEB3 or CPEB4 for their dendritic localization. This is an interesting result, since both these CPEB-family members were shown to be required for synaptic plasticity and dendritic spine changes in Aplysia and mouse, through a prion-like mechanism which confers these proteins the ability to form dynamic aggregates (Fioriti et al., 2015), and which does not appear to be involved in BDNF mRNA trafficking. Another peculiar aspect of CPEB3 is that it has been recognized as self-cleaving ribozyme throughout the evolution (Salehi-Ashtiani et al., 2006), which may explain why we and others (Morgan et al., 2010) found low cellular levels of this mRNA by PCR, in front of clearly detectable protein levels by western-blot and immunofluorescence.

We provide conclusive *in vitro* and *in vivo* evidence that *BDNF* transcripts with a short 3′ UTR can be targeted to dendrites in an activity-dependent manner by binding to CPEB-1, as previously suggested by two different laboratories (Oe and Yoneda, 2010; Baj et al., 2011). In addition, we show here that inducible dendritic targeting of the 3′ UTR short requires also ELAV-2 and -4. Interaction between the two ELAV sites on the short 3′ UTR may explain why the absence of either ELAV-2 or ELAV-4 leads to disruption of mRNA short targeting in dendrites. It has been suggested that ELAV-2-4 dimerization may allow the formation of RNA transporting granules able to interact with the cytoskeleton for transport and with the translational machinery (Gao and Keene, 1996; Antic and Keene, 1998). Moreover, we found one highly conserved CPEB-1 binding site also in the terminal part of long 3′ UTR, in close proximity to several motifs recognized by ELAV proteins; this region resulted to be a novel inducible DTE. Even if AU-rich binding sites were found in the long 3′ UTR, silencing of hnRNPA2/B1 did not affect long 3′ UTR mRNA trafficking most likely because of compensatory binding by hnRNP CBF-A (CArG Box binding Factor A), which was previously shown to be involved in BDNF mRNA trafficking (Raju et al., 2011). In the mid region of the long 3′ UTR, we identified a previously unknown retention signal able to overcome the constitutive dendritic targeting signal in the CDS mediated by Translin. These findings are in accordance with the observed increase in *BDNF* mRNA in dendrites of FMR1 knock-out mice under resting conditions (Louhivuori et al., 2011) which might account for an increased susceptibility to epileptic seizures in these animals (El Idrissi et al., 2005). Notably, FMR1 KO mice were found to have an exaggerated mTOR and PI3K activation (Sharma et al., 2010). Since mRNA transport in dendrites can be mediated by PI3K (Righi et al., 2000), it is conceivable that the lack of targeting repression by FMRP and the enhanced PI3K signaling may provide a synergistic effect on *BDNF* mRNA transport. In contrast, the dynamics of short 3′ UTR localization seem to rely on the activation of dormant targeting elements and proteins, apparently without a soma-retention mechanism. Interestingly, a similar organization of targeting elements has been observed in CaMKIIα a prominent dendritically localized transcript. In fact, two opposing localization signals have been identified at the level of *CaMKIIα* 3′ UTR: a DTE in the first 28–56 nts of 3′ UTR, promoting RNA dendritic trafficking (also known as the CNDLE sequence), that is overridden by a downstream retention signal whose inhibition is relieved after KCl-induced depolarization (Mori et al., 2000). In *CaMKIIα* mRNA, two CPE elements in *CaMKIIα* 3′ UTR seem to facilitate mRNA transport and translational activation (Huang et al., 2002). In addition, it has been reported that the presence of a translin binding site at the level of the coding region, is necessary for transport of *CaMKIIα* mRNA in dendrites (Severt et al., 1999), as for *BDNF* mRNA. In *BDNF* 3′ UTR mRNA, we found AU-rich regions, ELAV binding sites and a G-quartet-like structure similar to CaMKIIα (for comparison, see Dictenberg et al., 2008). Finally, *CaMKIIα* mRNA might be alternatively polyadenylated to generate a short or a long 3′ UTR, with the latter being much more abundant than the former. This striking parallelism between *BDNF* and *CaMKIIα cis* and *trans*-elements involved in their activity-dependent subcellular sorting further supports the “RNA operon theory” (Keene and Tenenbaum, 2002), by which common signals and RBPs are shared by different mRNAs with common functions (e.g., synaptic plasticity and potentiation).

The results shown here, together with previous findings from our laboratory, provide strong evidence that *BDNF* mRNAs display both activity-dependence and transcript selectivity. Transcript selectivity is mediated by signals located in the 5′ UTRs that for some variants may be either permissive to distal dendritic targeting or, in other variants, may promote mRNA retention in the soma/proximal dendrites by overriding localization signals harbored in the CDS and 3′ UTRs (Chiaruttini et al., 2009). These findings, together with the role of both 3′ UTRs in activity-dependent dendritic localization, as shown here, strongly argue in favor of a mechanism of BDNF mRNA subcellular sorting regulated by a tripartite signal (5′ UTRs = selectors, CDS = constitutive DTE, and 3′ UTRs = inducible DTE). In conclusion, we propose a model in which inducible dendritic targeting signals are harbored in both 3′ UTRs which respond selectively to different extracellular stimuli, thus proving the basis for a differential regulation of BDNF variants for the fine tuning of distal dendritic compartment plasticity.

## Conflict of Interest Statement

The authors declare that the research was conducted in the absence of any commercial or financial relationships that could be construed as a potential conflict of interest.

## References

[B1] Aid T. Kazantseva A. Piirsoo M. Palm K. Timmusk T. (2007). Mouse and rat BDNF gene structure and expression revisited. *J. Neurosci. Res.* 85 525–535. 10.1002/jnr.21139 17149751 PMC1878509

[B2] Aliaga E. E. Mendoza I. Tapia-Arancibia L. (2009). Distinct subcellular localization of BDNF transcripts in cultured hypothalamic neurons and modification by neuronal activation. *J. Neural Transm.* 116 23–32. 10.1007/s00702-008-0159-8 19082527

[B3] Allen M. Bird C. Feng W. Liu G. Li W. Perrone-Bizzozero N. I. (2013). HuD promotes BDNF expression in brain neurons via selective stabilization of the BDNF long 3’UTR mRNA. *PLoS ONE* 8:e55718 10.1371/journal.pone.0055718 PMC3561324 23383270

[B4] An J. J. Gharami K. Liao G. Y. Woo N. H. Lau A. G. Vanevski F. (2008). Distinct role of long 3’ UTR BDNF mRNA in spine morphology and synaptic plasticity in hippocampal neurons. *Cell* 134 175–187. 10.1016/j.cell.2008.05.045 18614020 PMC2527207

[B5] Antic D. Keene J. D. (1998). Messenger ribonucleoprotein complexes containing human ELAV proteins: interactions with cytoskeleton and translational apparatus. *J. Cell Sci.* 111(Pt 2), 183–197. 9405302 10.1242/jcs.111.2.183

[B6] Baj G. Del Turco D. Schlaudraff J. Torelli L. Deller T. Tongiorgi E. (2013). Regulation of the spatial code for BDNF mRNA isoforms in the rat hippocampus following pilocarpine-treatment: a systematic analysis using laser microdissection and quantitative real-time PCR. *Hippocampus* 23 413–423. 10.1002/hipo.22100 23436435

[B7] Baj G. Leone E. Chao M. V. Tongiorgi E. (2011). Spatial segregation of BDNF transcripts enables BDNF to differentially shape distinct dendritic compartments. *Proc. Natl. Acad. Sci. U.S.A.* 108 16813–16818. 10.1073/pnas.1014168108 21933955 PMC3189043

[B8] Bernd P. (2008). The role of neurotrophins during early development. *Gene Expr.* 14 241–250. 10.3727/105221608786883799 19110723 PMC6042000

[B9] Chiaruttini C. Sonego M. Baj G. Simonato M. Tongiorgi E. (2008). BDNF mRNA splice variants display activity-dependent targeting to distinct hippocampal laminae. *Mol. Cell. Neurosci.* 37 11–19. 10.1016/j.mcn.2007.08.011 17919921

[B10] Chiaruttini C. Vicario A. Li Z. Baj G. Braiuca P. Wu Y. (2009). Dendritic trafficking of BDNF mRNA is mediated by translin and blocked by the G196A (Val66Met) mutation. *Proc. Natl. Acad. Sci. U.S.A.* 106 16481–16486. 10.1073/pnas.0902833106 19805324 PMC2752540

[B11] Darnell J. C. Jensen K. B. Jin P. Brown V. Warren S. T. Darnell R. B. (2001). Fragile X mental retardation protein targets G quartet mRNAs important for neuronal function. *Cell* 107 489–499. 10.1016/S0092-8674(01)00566-9 11719189

[B12] Davidovic L. Navratil V. Bonaccorso C. M. Catania M. V. Bardoni B. Dumas M. E. (2011). A metabolomic and systems biology perspective on the brain of the fragile X syndrome mouse model. *Genome Res.* 21 2190–2202. 10.1101/gr.116764.110 21900387 PMC3227107

[B13] Dictenberg J. B. Swanger S. A. Antar L. N. Singer R. H. Bassell G. J. (2008). A direct role for FMRP in activity-dependent dendritic mRNA transport links filopodial-spine morphogenesis to fragile X syndrome. *Dev. Cell* 14 926–939. 10.1016/j.devcel.2008.04.003 18539120 PMC2453222

[B14] Edbauer D. Neilson J. R. Foster K. A. Wang C. F. Seeburg D. P. Batterton M. N. (2010). Regulation of synaptic structure and function by FMRP-associated microRNAs miR-125b and miR-132. *Neuron* 65 373–384. 10.1016/j.neuron.2010.01.005 20159450 PMC5018398

[B15] El Idrissi A. Ding X. H. Scalia J. Trenkner E. Brown W. T. Dobkin C. (2005). Decreased GABA(A) receptor expression in the seizure-prone fragile X mouse. *Neurosci. Lett.* 377 141–146. 10.1016/j.neulet.2004.11.087 15755515

[B16] Fioriti L. Myers C. Huang Y. Y. Li X. Stephan J. S. Trifilieff P. (2015). The persistence of hippocampal-based memory requires protein synthesis mediated by the prion-like protein CPEB3. *Neuron* 86 1433–1448. 10.1016/j.neuron.2015.05.021 26074003

[B17] Fukuchi M. Tsuda M. (2010). Involvement of the 3’-untranslated region of the brain-derived neurotrophic factor gene in activity-dependent mRNA stabilization. *J. Neurochem.* 115 1222–1233. 10.1111/j.1471-4159.2010.07016.x 20874756

[B18] Gao F. B. Keene J. D. (1996). Hel-N1/Hel-N2 proteins are bound to poly(A)+ mRNA in granular RNP structures and are implicated in neuronal differentiation. *J. Cell Sci.* 109(Pt 3), 579–589. 8907704 10.1242/jcs.109.3.579

[B19] Gharami K. Das S. (2014). BDNF local translation in viable synaptosomes: implication in spine maturation. *Neurochem. Int.* 69 28–34. 10.1016/j.neuint.2014.02.009 24632004

[B20] Gorski J. A. Zeiler S. R. Tamowski S. Jones K. R. (2003). Brain-derived neurotrophic factor is required for the maintenance of cortical dendrites. *J. Neurosci.* 23 6856–6865. 12890780 10.1523/JNEUROSCI.23-17-06856.2003 PMC6740724

[B21] Huang Y. S. Jung M. Y. Sarkissian M. Richter J. D. (2002). N-methyl-D-aspartate receptor signaling results in Aurora kinase-catalyzed CPEB phosphorylation and alpha CaMKII mRNA polyadenylation at synapses. *EMBO J.* 21 2139–2148. 10.1093/emboj/21.9.2139 11980711 PMC125376

[B22] Huang Y. S. Kan M. C. Lin C. L. Richter J. D. (2006). CPEB3 and CPEB4 in neurons: analysis of RNA-binding specificity and translational control of AMPA receptor GluR2 mRNA. *EMBO J.* 25 4865–4876. 10.1038/sj.emboj.7601322 17024188 PMC1618119

[B23] Keene J. D. Tenenbaum S. A. (2002). Eukaryotic mRNPs may represent posttranscriptional operons. *Mol. Cell* 9 1161–1167. 10.1016/S1097-2765(02)00559-2 12086614

[B24] Knowles R. B. Kosik K. S. (1997). Neurotrophin-3 signals redistribute RNA in neurons. *Proc. Natl. Acad. Sci. U.S.A.* 94 14804–14808. 10.1073/pnas.94.26.14804 9405694 PMC25118

[B25] Ladd A. N. Charlet N. Cooper T. A. (2001). The CELF family of RNA binding proteins is implicated in cell-specific and developmentally regulated alternative splicing. *Mol. Cell. Biol.* 21 1285–1296. 10.1128/MCB.21.4.1285-1296.2001 11158314 PMC99581

[B26] Leal G. Comprido D. Duarte C. B. (2014). BDNF-induced local protein synthesis and synaptic plasticity. *Neuropharmacology* 76(Pt C), 639–656. 10.1016/j.neuropharm.2013.04.005 23602987

[B27] Lein E. S. Hohn A. Shatz C. J. (2000). Dynamic regulation of BDNF and NT-3 expression during visual system development. *J. Comp. Neurol.* 420 1–18. 10.1002/(SICI)1096-9861(20000424)420:1<1::AID-CNE1>3.0.CO;2-H 10745216

[B28] Lim C. S. Alkon D. L. (2012). Protein kinase C stimulates HuD-mediated mRNA stability and protein expression of neurotrophic factors and enhances dendritic maturation of hippocampal neurons in culture. *Hippocampus* 22 2303–2319. 10.1002/hipo.22048 22736542

[B29] Lindholm D. Da Penha Berzaghi M. Cooper J. Thoenen H. Castren E. (1994). Brain-derived neurotrophic factor and neurotrophin-4 increase neurotrophin-3 expression in the rat hippocampus. *Int. J. Dev. Neurosci.* 12 745–751. 10.1016/0736-5748(94)90054-X 7747601

[B30] Louhivuori V. Vicario A. Uutela M. Rantamaki T. Louhivuori L. M. Castren E. (2011). BDNF and TrkB in neuronal differentiation of Fmr1-knockout mouse. *Neurobiol. Dis.* 41 469–480. 10.1016/j.nbd.2010.10.018 21047554

[B31] Martin K. C. Ephrussi A. (2009). mRNA localization: gene expression in the spatial dimension. *Cell* 136 719–730. 10.1016/j.cell.2009.01.044 19239891 PMC2819924

[B32] McAllister A. K. Katz L. C. Lo D. C. (1997). Opposing roles for endogenous BDNF and NT-3 in regulating cortical dendritic growth. *Neuron* 18 767–778. 10.1016/S0896-6273(00)80316-5 9182801

[B33] Moraes K. C. Wilusz C. J. Wilusz J. (2006). CUG-BP binds to RNA substrates and recruits PARN deadenylase. *RNA* 12 1084–1091. 10.1261/rna.59606 16601207 PMC1464848

[B34] Morgan M. Iaconcig A. Muro A. F. (2010). CPEB2, CPEB3 and CPEB4 are coordinately regulated by miRNAs recognizing conserved binding sites in paralog positions of their 3’-UTRs. *Nucleic Acids Res.* 38 7698–7710. 10.1093/nar/gkq635 20660482 PMC2995058

[B35] Mori D. Sasagawa N. Kino Y. Ishiura S. (2008). Quantitative analysis of CUG-BP1 binding to RNA repeats. *J. Biochem.* 143 377–383. 10.1093/jb/mvm230 18039683

[B36] Mori Y. Imaizumi K. Katayama T. Yoneda T. Tohyama M. (2000). Two cis-acting elements in the 3’ untranslated region of alpha-CaMKII regulate its dendritic targeting. *Nat. Neurosci.* 3 1079–1084. 10.1038/80591 11036263

[B37] Oe S. Yoneda Y. (2010). Cytoplasmic polyadenylation element-like sequences are involved in dendritic targeting of BDNF mRNA in hippocampal neurons. *FEBS Lett.* 584 3424–3430. 10.1016/j.febslet.2010.06.040 20603120

[B38] Paillard L. Legagneux V. Beverley Osborne H. (2003). A functional deadenylation assay identifies human CUG-BP as a deadenylation factor. *Biol. Cell* 95 107–113. 10.1016/S0248-4900(03)00010-8 12799066

[B39] Pattabiraman P. P. Tropea D. Chiaruttini C. Tongiorgi E. Cattaneo A. Domenici L. (2005). Neuronal activity regulates the developmental expression and subcellular localization of cortical BDNF mRNA isoforms in vivo. *Mol. Cell. Neurosci.* 28 556–570. 10.1016/j.mcn.2004.11.010 15737745

[B40] Patz S. Wahle P. (2006). Developmental changes of neurotrophin mRNA expression in the layers of rat visual cortex. *Eur. J. Neurosci.* 24 2453–2460. 10.1111/j.1460-9568.2006.05126.x 17100834

[B41] Philips A. V. Timchenko L. T. Cooper T. A. (1998). Disruption of splicing regulated by a CUG-binding protein in myotonic dystrophy. *Science* 280 737–741. 10.1126/science.280.5364.737 9563950

[B42] Pruunsild P. Kazantseva A. Aid T. Palm K. Timmusk T. (2007). Dissecting the human BDNF locus: bidirectional transcription, complex splicing, and multiple promoters. *Genomics* 90 397–406. 10.1016/j.ygeno.2007.05.004 17629449 PMC2568880

[B43] Raju C. S. Fukuda N. Lopez-Iglesias C. Goritz C. Visa N. Percipalle P. (2011). In neurons, activity-dependent association of dendritically transported mRNA transcripts with the transacting factor CBF-A is mediated by A2RE/RTS elements. *Mol. Biol. Cell* 22 1864–1877. 10.1091/mbc.E10-11-0904 21471000 PMC3103402

[B44] Ratti A. Fallini C. Cova L. Fantozzi R. Calzarossa C. Zennaro E. (2006). A role for the ELAV RNA-binding proteins in neural stem cells: stabilization of Msi1 mRNA. *J. Cell Sci.* 119 1442–1452. 10.1242/jcs.02852 16554442

[B45] Righi M. Tongiorgi E. Cattaneo A. (2000). Brain-derived neurotrophic factor (BDNF) induces dendritic targeting of BDNF and tyrosine kinase B mRNAs in hippocampal neurons through a phosphatidylinositol-3 kinase-dependent pathway. *J. Neurosci.* 20 3165–3174. 10777780 10.1523/JNEUROSCI.20-09-03165.2000 PMC6773127

[B46] Rodriguez A. J. Czaplinski K. Condeelis J. S. Singer R. H. (2008). Mechanisms and cellular roles of local protein synthesis in mammalian cells. *Curr. Opin. Cell Biol.* 20 144–149. 10.1016/j.ceb.2008.02.004 18378131 PMC2404115

[B47] Rozhdestvensky T. S. Kopylov A. M. Brosius J. Huttenhofer A. (2001). Neuronal BC1 RNA structure: evolutionary conversion of a tRNA(Ala) domain into an extended stem-loop structure. *RNA* 7 722–730. 10.1017/S1355838201002485 11350036 PMC1370124

[B48] Salehi-Ashtiani K. Luptak A. Litovchick A. Szostak J. W. (2006). A genomewide search for ribozymes reveals an HDV-like sequence in the human CPEB3 gene. *Science* 313 1788–1792. 10.1126/science.1129308 16990549

[B49] Savkur R. S. Philips A. V. Cooper T. A. (2001). Aberrant regulation of insulin receptor alternative splicing is associated with insulin resistance in myotonic dystrophy. *Nat. Genet.* 29 40–47. 10.1038/ng704 11528389

[B50] Severt W. L. Biber T. U. Wu X. Hecht N. B. Delorenzo R. J. Jakoi E. R. (1999). The suppression of testis-brain RNA binding protein and kinesin heavy chain disrupts mRNA sorting in dendrites. *J. Cell Sci.* 112(Pt 21), 3691–3702. 10523505 10.1242/jcs.112.21.3691

[B51] Sharma A. Hoeffer C. A. Takayasu Y. Miyawaki T. Mcbride S. M. Klann E. (2010). Dysregulation of mTOR signaling in fragile X syndrome. *J. Neurosci.* 30 694–702. 10.1523/JNEUROSCI.3696-09.2010 20071534 PMC3665010

[B52] Siepel A. Bejerano G. Pedersen J. S. Hinrichs A. S. Hou M. Rosenbloom K. (2005). Evolutionarily conserved elements in vertebrate, insect, worm, and yeast genomes. *Genome Res.* 15 1034–1050. 10.1101/gr.3715005 16024819 PMC1182216

[B53] Soule J. Messaoudi E. Bramham C. R. (2006). Brain-derived neurotrophic factor and control of synaptic consolidation in the adult brain. *Biochem. Soc. Trans.* 34 600–604. 10.1042/BST0340600 16856871

[B54] Steward O. Schuman E. M. (2001). Protein synthesis at synaptic sites on dendrites. *Annu. Rev. Neurosci.* 24 299–325. 10.1146/annurev.neuro.24.1.299 11283313

[B55] Timchenko N. A. Welm A. L. Lu X. Timchenko L. T. (1999). CUG repeat binding protein (CUGBP1) interacts with the 5’ region of C/EBPbeta mRNA and regulates translation of C/EBPbeta isoforms. *Nucleic Acids Res.* 27 4517–4525. 10.1093/nar/27.22.4517 10536163 PMC148737

[B56] Timmusk T. Palm K. Metsis M. Reintam T. Paalme V. Saarma M. (1993). Multiple promoters direct tissue-specific expression of the rat BDNF gene. *Neuron* 10 475–489. 10.1016/0896-6273(93)90335-O 8461137

[B57] Tongiorgi E. (2008). Activity-dependent expression of brain-derived neurotrophic factor in dendrites: facts and open questions. *Neurosci. Res.* 61 335–346. 10.1016/j.neures.2008.04.013 18550187

[B58] Tongiorgi E. Armellin M. Giulianini P. G. Bregola G. Zucchini S. Paradiso B. (2004). Brain-derived neurotrophic factor mRNA and protein are targeted to discrete dendritic laminas by events that trigger epileptogenesis. *J. Neurosci.* 24 6842–6852. 10.1523/JNEUROSCI.5471-03.2004 15282290 PMC6729709

[B59] Tongiorgi E. Righi M. Cattaneo A. (1997). Activity-dependent dendritic targeting of BDNF and TrkB mRNAs in hippocampal neurons. *J. Neurosci.* 17 9492–9505. 9391005 10.1523/JNEUROSCI.17-24-09492.1997 PMC6573421

[B60] Tongiorgi E. Righi M. Cattaneo A. (1998). A non-radioactive in situ hybridization method that does not require RNAse-free conditions. *J. Neurosci. Methods* 85 129–139. 10.1016/S0165-0270(98)00123-X 9874149

[B61] Verpelli C. Piccoli G. Zibetti C. Zanchi A. Gardoni F. Huang K. (2010). Synaptic activity controls dendritic spine morphology by modulating eEF2-dependent BDNF synthesis. *J. Neurosci.* 30 5830–5842. 10.1523/JNEUROSCI.0119-10.2010 20427644 PMC6632604

[B62] Wickens M. Bernstein D. S. Kimble J. Parker R. (2002). A PUF family portrait: 3’UTR regulation as a way of life. *Trends Genet.* 18 150–157. 10.1016/S0168-9525(01)02616-6 11858839

[B63] Will T. J. Tushev G. Kochen L. Nassim-Assir B. Cajigas I. J. Tom Dieck S. (2013). Deep sequencing and high-resolution imaging reveal compartment-specific localization of Bdnf mRNA in hippocampal neurons. *Sci. Signal.* 6:rs16 10.1126/scisignal.2004520 PMC5321484 24345682

[B64] Zhang H. L. Singer R. H. Bassell G. J. (1999). Neurotrophin regulation of beta-actin mRNA and protein localization within growth cones. *J. Cell Biol.* 147 59–70. 10.1083/jcb.147.1.59 10508855 PMC2164987

