# Supplementary material for: Dendritic targeting of short and long 3′ UTR BDNF mRNA is regulated by BDNF or NT-3 and distinct sets of RNA-binding proteins
Source: Front Mol Neurosci. 2015 Oct 29;8:62. doi: 10.3389/fnmol.2015.00062 (PMC4624863; doi:10.3389/fnmol.2015.00062)
Supplement: Supplementary file 1 [file Data_Sheet_1.DOCX]

***Supplementary Material***

**Short and long 3’UTR BDNF mRNA dendritic targeting is induced by BDNF or NT-3, respectively using distinct sets of RNA-binding proteins**

**Abbreviated title:** Short and long BDNF mRNA inducible dendritic targeting

**Authors:**

Annalisa Vicario^1^, Andrea Colliva^1^, Antonia Ratti^2,3^, Laetitia Davidovic^4^, Gabriele Baj^1^, Łukasz Gricman^1^, Claudia Colombrita^2,3^, Alberto Pallavicini^1^, Kevin R. Jones^5^, Barbara Bardoni^4^ and Enrico Tongiorgi^1*^

**Affiliations:**

^1^ Department of Life Sciences, University of Trieste, Trieste, Italy

^2^ Laboratory of Neuroscience - IRCCS Istituto Auxologico Italiano, Milano, Italy

^3^ Department of Pathophysiology and Transplantation, “Dino Ferrari Center”, Università degli Studi di Milano,Milano, Italy

^4^ INSERM, IPMC - CNRS UMR7275, Valbonne, France

^5^ Department of Molecular Cellular and Developmental Biology, University of Colorado, Boulder,Colorado, USA

*** Corresponding author**:

Prof. Enrico Tongiorgi

University of Trieste

Department of Life Sciences

Via Giorgieri, 5 - Building Q

34127 Trieste – Italy
tel: +39 040 558 8724 - Fax: +39 040 558 3133;

mail: [tongi@units.it](mailto:tongi@units.it)

**Supplementary figure 1**





**Supplementary Figure S1**. A) *c-fos* immunostaining on SK-N-BE cells untreated (control) or treated with KCl 10 mM. B) *c-fos* immunostaining on SK-N-BE cells upont treatment with different neurotrophins (NGF, BDNF, NT-3 and NT-4) at 50 ng/mL for 3 hours. Scale bar, 10µm.

**Supplementary figure S2**


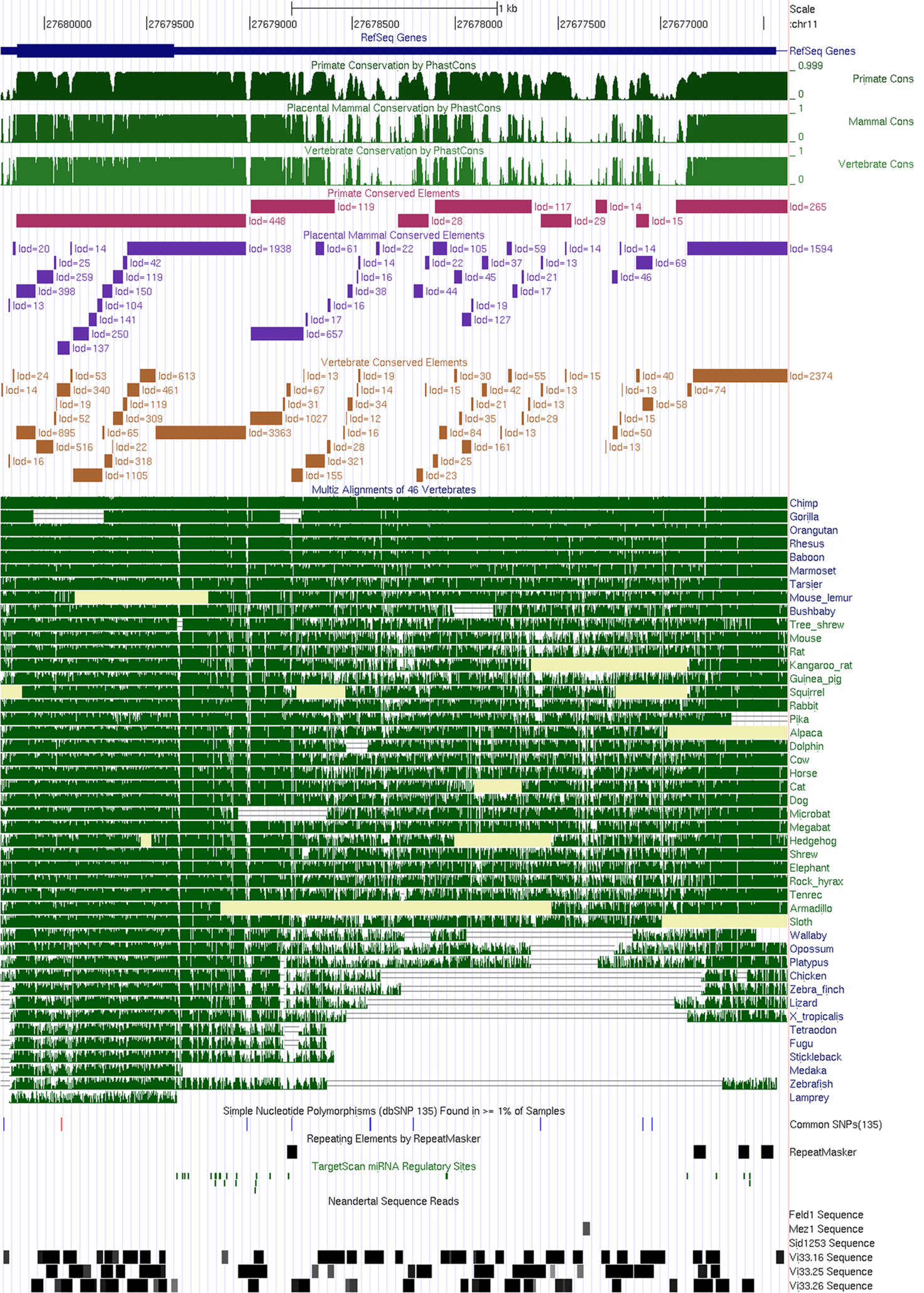


**Supplementary Figure S2**. Bioinformatic analysis using phastCons showing the degree of consensus on BDNF coding regions and 3’UTR among Primates (top), Mammals (middle) and vertebrates (bottom). Similarity scores range from 0 (= no homology) to 1 (identical sequence). Numbers at the top of the figure indicate the nucleotide position along the *BDNF* gene along chromosome 11.

**Supplementary figure S3**


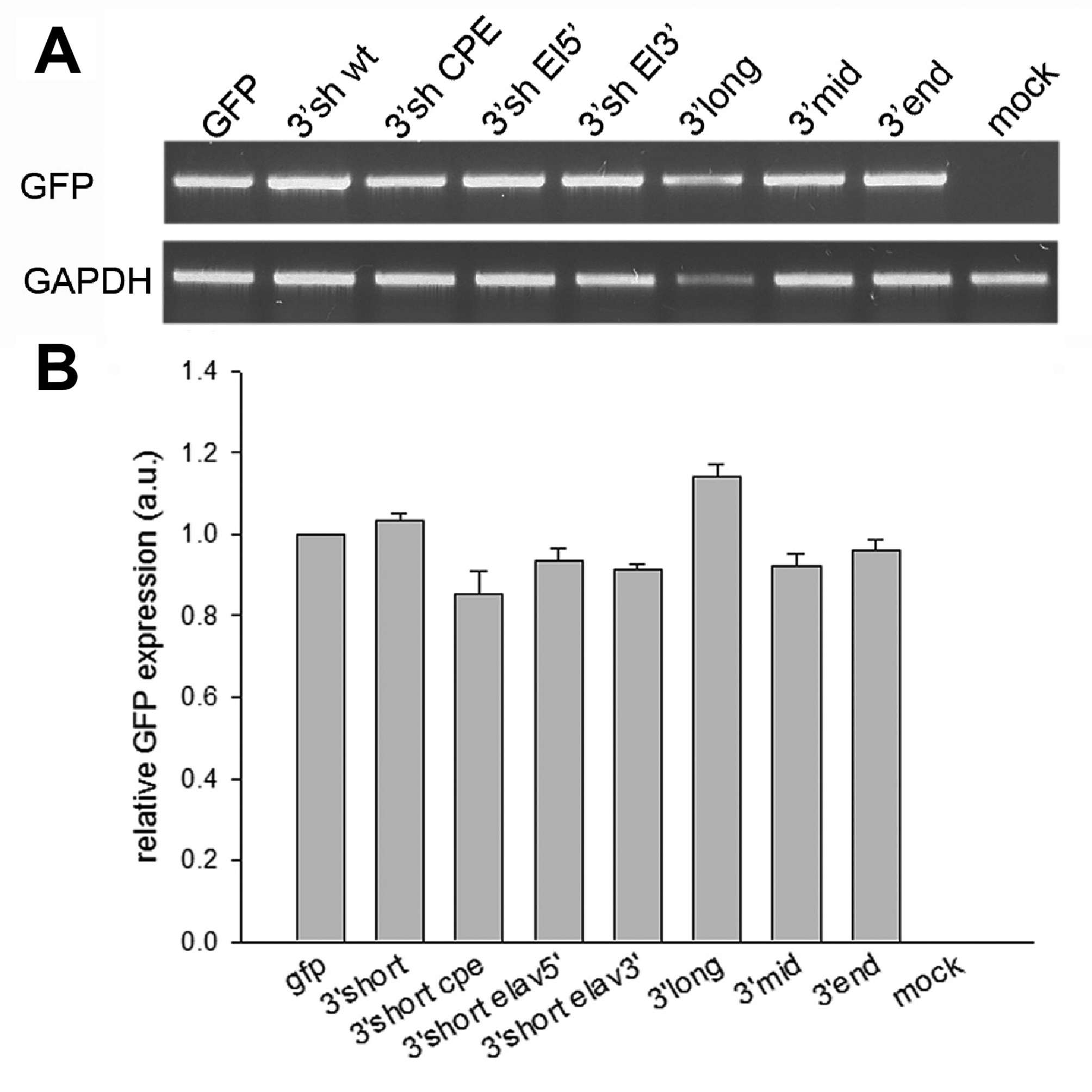


**Supplementary figure S3.** GFP-3'UTRs constructs stability. A) Representative RT-PCRs of GFP and different GFP-3'UTRs’ chimeras constructs to evaluate mRNA expression levels in cultured neurons. *GADPH* expression level has been used as control for normalization. B) Quantification of the expression levels of the different constructs. *GAPDH* was used for normalization.

**Supplementary Table S1.** Primers and PCR conditions used to generate plasmids and probes used in the assays.

|  | **Primer Forward** | **Primer Reverse** | **PCR CONDITIONS** | | | | | |
| --- | --- | --- | --- | --- | --- | --- | --- | --- |
| 3’UTR short | GGGCGGCCGCTGGATTTATGTTGTATAG | GTCAGTTAACTTTATTATCAATTCACAATTAAAGC | DEN | 98°C | | 10 “ | | 31 cycles |
|  |  |  | ANN | 56°C | | 20” | |  |
|  |  |  | EXT | 72°C | | 30 “ | |  |
| 3’UTR long | GGGCGGCCGCTGGATTTATGTTGTATAG | GCCGGTTAACTTACAATAGGCTTCTGATGTGG | DEN | 98°C | | 10 “ | | 31 cycles |
|  |  |  | ANN | 56°C | | 20” | |  |
|  |  |  | EXT | 72°C | | 90 “ | |  |
| 3’UTRmid | actaGCGGCCGCagcacattctttccctcc | GTCAGTTAACgatagcatcactgctatgcc | DEN | 98°C | | 10 “ | | 31 cycles |
|  |  |  | ANN | 56°C | | 20” | |  |
|  |  |  | EXT | 72°C | | 30 “ | |  |
| 3’UTR end | gtcagcggccgccaatctcacggtattcgaagggc | GCCGGTTAACTTACAATAGGCTTCTGATGTGG | DEN | 98°C | | 10 “ | | 31 cycles |
|  |  |  | ANN | 56°C | | 20” | |  |
|  |  |  | EXT | 72°C | | 30 “ | |  |
| **PCR Assembly 1st round** | | | | | | | |  |
| 3’UTR short mut CPE | TgCAGCGGCCGCtggatttatgttgtatagattatattgagacaaaaattatctatttgtatatatacataacaggg | acatgcagttccgatcgttatctttttcttaactgaataatttaccctgttatgtatatatac | DEN | 98°C | | 10 “ | | 31 cycles |
|  |  |  | ANN | 57.5°C | | 120 “ | |  |
|  |  |  | EXT | 72°C | | 30 “ | |  |
| 3’UTR short mutELAVdw | TgCAGCGGCCGCtggatttatgttgtatagattatattgagacaaaaattatctatttgtatatatacataacaggg | ggtcatggatatgtccgcgcgctagattgtag | DEN | 98°C | | 10 “ | | 31 cycles |
|  |  |  | ANN | 57.5°C | | 120 “ | |  |
|  |  |  | EXT | 72°C | | 30 “ | |  |
| **PCR Assembly 2nd round** | | | | | | | |  |
| 3’UTRshort-mutCPE or 3’UTRshort-mutELAVdw from 1^st^ PCR round | | | DEN | 98°C | | 10“ | | 31 cycles |
|  |  |  | ANN | 58°C | | 120“ | |  |
|  |  |  | EXT | 72°C | | 30“ | |  |
| PCR fragments were then digested with NotI/HpaI (New England Biolabs) and ligated into pEGFPN1 vector (Clontech), Abbrev. DEN = denaturation, ANN = annealing, EXT = extention | | | | | | | | |
| **RT-PCR on CLIP experiments** | | | | | | | | |
| BDNF coding sequence | GAAGTAAACGTCCACGGACAA | GATGTCGTCGTCAGACCTCTC | DEN | | 95°C | | 30“ | 40 cycles |
|  |  |  | ANN | | 56°C | | 10“ |  |
|  |  |  | EXT | | 72°C | | 30“ |  |

**Supplementary Table S2.** Primers containing T7 promoter used to generate dsRNA for RBPs.

|  | **Primer Forward** | **Primer Reverse** |
| --- | --- | --- |
| **CPEB-1** | GCGTAATACGACTCACTATAGGGtctgacaggctgggaccgac | GCGTAATACGACTCACTATAGGGtgaagccactggtgtctgag |
| **CPEB-2** | GCGTAATACGACTCACTATAGGGtggggagcaatgcatggcagag | GCGTAATACGACTCACTATAGGGaacagattgtcggttcctgg |
| **CPEB-3** | GCGTAATACGACTCACTATAGGGtcctgcgcaaaccatgcagg | GCGTAATACGACTCACTATAGGGtgcctccgaagaccgggttgac |
| **CPEB-4** | GCGTAATACGACTCACTATAGGGagtggaacggtattcccgcaagg | GCGTAATACGACTCACTATAGGGagacgcctccatacagccggtc |
| **ELAV-1** | GCGTAATACGACTCACTATAGGGtccctcagaacatgacccagg | GCGTAATACGACTCACTATAGGGgcaaacttcactgtgatgggctc |
| **ELAV-2** | GCGTAATACGACTCACTATAGGGttgtcgaccaggtcactggc | GCGTAATACGACTCACTATAGGGttgtcgaccaggtcactggc |
| **ELAV-3** | GCGTAATACGACTCACTATAGGGagccactgacgacagcaagacc | GCGTAATACGACTCACTATAGGGctggctcatggtcttggggag |
| **ELAV-4** | GCGTAATACGACTCACTATAGGGtcaggagtctctttgggagcattgg | GCGTAATACGACTCACTATAGGGtgggcttctggccattcagc |
| **Translin** | GCGTAATACGACTCACTATAGGGtgagcactggaggtttgtgc | GCGTAATACGACTCACTATAGGGcgtagcgtttcctcagggag |
| **hnRNPA2/B1** | gcgtaatacgactcactataggggaagatactgaggaacatcacc | gcgtaatacgactcactatagggcctggtccgaaatttccaccgcca |
| **scramble** | gcgtaatacgactcactatagggcggccatgattggggtgcttg | gcgtaatacgactcactatagggactaacgggatttcacgaggcca |
| **PROTOCOL** | | |
| The target regions were amplified from whole rat brain and testis cDNA by PCR at 94 °C for 1 min, 55 °C for 1 min, 72 °C for 2 min (30 cycles), and 72 °C for 10 min. PCR products were gel-purified and used as template for *in vitro* transcription using T7 RNA polymerase and the *Silencer* siRNA mixture kit (Ambion). Following DNase and RNase A treatment for 1 h, double stranded RNA was purified through spin columns (Promega) and digested with RNase III according to the manufacturer’s instructions to yield siRNA mixtures. The mixtures of 12–30-bp double-stranded RNAs contain 5’-PO_4_, 3’-OH, and 2-nucleotide 3’overhangs similar to siRNA produced *in vivo*. For FMR1 and FXR2 the following RNA oligos were purchased (SIGMA) and annealed to generate siRNAs in vitro | | |
| **FMR1-siRNA** | GGUUUAUUCCAGAGCAAAUtt | GCAUGUGAUGCUACGUAUAtt |
| **FXR2 siRNA** | AAGUAGUCGAGUGCGUGGAUUtt | gcuaccuugaauuuuccgatt |

**Supplementary Table S3**. List and conditions of antibodies used

| **Primary Antibodies** | | | |
| --- | --- | --- | --- |
| **Target** | **Source and code** | **Dilution** | **Conditions** |
| **Anti Dig-AP** | Roche 11093274910 | 1:1000 for ISH | 3 hours at RT |
| **Anti Dig-HRP** | Roche 11207733910 | 1:300 for FISH | 1 hour at RT |
| **CPEB-1** | Abcam AB73287 | 1:50 for FISH  1:500 for WB | 1.5 hours at RT  O/N at 4° |
| **CPEB-2** | Life Span LS-C31563  Abcam AB51069 | 1:50 for FISH  1:400 for WB | 1.5 hours at RT  O/N at 4° |
| **CPEB-3** | Abcam AB10883 | 1:50 for FISH  1:700 for WB | 1.5 hours at RT  O/N at 4° |
| **CPEB-4** | Abcam AB83009 | 1:50 for FISH  1:500 for WB | 1.5 hours at RT  O/N at 4° |
| **ELAV-1** | Abcam Ab85539 | 1:50 for FISH  1:500 for WB | 1.5 hours at RT  O/N at 4° |
| **ELAV-2** | Genetex GTX12030  Abcam AB12030 | 1:200 for FISH  1:500 for WB | 1.5 hours at RT  O/N at 4° |
| **ELAV-3** | Santacruz AC26421 | 1:50 for FISH  1:1000 for WB | 1.5 hours at RT  O/N at 4° |
| **ELAV-4** | Abcam AB14369 | 1:100 for FISH  1:1000 for WB | 1.5 hours at RT  O/N at 4° |
| **FMRP** | Abcam AB69815 | 1:100 for FISH  1:1000 for WB | 1.5 hours at RT  O/N at 4° |
| **FXR2** | Abcam AB65122 | 1:100 for FISH  1:700 for WB | 1.5 hours at RT  O/N at 4° |
| **hnRNPA2/B1** | Abcam AB6102 | 1:1000 for WB | O/N at 4° |
| **Translin** | (kind gift of prof. Baraban) | 1:200 for WB | O/N at 4° |
| **BDNF** | Santa Cruz SC546 | 1:100 for WB | 48 hours at 4° |
| **GFP** | Clonetech 632375 | 1:4000 for WB | O/N at 4° |
| **Alpha-Tubulin** | Sigma T6074 | 1:20000 for WB | 1 hour at RT ° |
| **Secondary Antibodies** | | | |
| **Target** | **Source and code** | **dilution** | **Conditions** |
| **Anti rabbit-HRP** | Dako P0448 | 1:400 for IHC | 3 hours at RT |
| **Anti rabbit-HRP** | Jackson 111035003 | 1:10000 for WB | 1 hours at RT |
| **Anti mouse-HRP** | Sigma A9044 | 1:10000 for WB | 1 hours at RT |
| **Anti goat-HRP** | Dako P0449 | 1:10000 for WB | 1 hours at RT |
| **Anti mouse-AP** | Dako R0270 | 1:1500 for WB | 1 hours at RT |
| **Anti rabbit-alexa488** | Life Technologies A11008 | 1:200 for FISH | 1 hours at RT |
| **Anti goat-alexa488** | Life Technologies A11055 | 1:200 for FISH | 1 hours at RT |

**Supplementary Table S4.** Densitometric evaluation of the semi-quantitative assays performed to evaluate siRNA specificity trough (reverse transcriptase-PCR, **A**) and efficacy (Western-Blot, **B**).

| **A** | | | | | | | | |
| --- | --- | --- | --- | --- | --- | --- | --- | --- |
| **RBP** | **CTRL** | | **si CPEB1** | **si CPEB2** | | **siCPEB1&2** | **siCPEB3** | **siCPEB4** |
| **CPEB1** | 100 % | | 34.9 % | 117.1 % | | 41.2 % | 124.9 % | 101.0 % |
| **CPEB2** | 100 % | | 122.8 % | 42.7 % | | 60.5 % | 138.5 % | 131.5 % |
| **CPEB3** | N.D. | | N.D. | N.D. | | N.D. | N.D. | N.D. |
| **CPEB4** | 100 % | | 126.1 % | 116.7 % | | 127.8 % | 103.8 % | 55.2 % |
| **RBP** | **CTRL** | | **si ELAV1** | **si ELAV2** | | **si ELAV3** | **si ELAV4** |  |
| **ELAV1** | 100 % | | 43.5 % | 113.3 % | | 148.9 % | 114.1 % |  |
| **ELAV2** | 100 % | | 113.6 % | 34.0 % | | 92.6 % | 89.1 % |  |
| **ELAV3** | 100 % | | 111.8 % | 110.2 % | | 52.9 % | 93.3 % |  |
| **ELAV4** | 100 % | | 102.7 % | 113.8 % | | 102.4 % | 27.3 % |  |
| **B** | | | | | | | | |
| **RBP** | | **CTRL** | | | **RNAi** | | | |
| **CPEB1** | | 100 % | | | 43.4 % | | | |
| **CPEB2** | | 100 % | | | 66.98 % | | | |
| **CPEB3** | | 100 % | | | 28.62 % | | | |
| **CPEB4** | | 100 % | | | 44.75 % | | | |
| **ELAV1** | | 100 % | | | 72.8 % | | | |
| **ELAV2** | | 100 % | | | 46.85 % | | | |
| **ELAV3** | | 100 % | | | 53.18 % | | | |
| **ELAV4** | | 100 % | | | 35.69 % | | | |
| **FMRP** | | 100 % | | | 59.52 % | | | |
| **FXR2** | | 100 % | | | 21.5 % | | | |
| **TRANSLIN** | | 100 % | | | 18.77 % | | | |
| **hnRNP A2/B1** | | 100 % | | | 45.5 % | | | |

All values represent the percentage of remaining signal vs control condition following the RNAi treatment.
